# Patterns of ecological specialization among microbial populations in the Red Sea and diverse oligotrophic marine environments

**DOI:** 10.1002/ece3.593

**Published:** 2013-05-11

**Authors:** Luke R Thompson, Chris Field, Tamara Romanuk, David Ngugi, Rania Siam, Hamza El Dorry, Ulrich Stingl

**Affiliations:** 1 Red Sea Research Center, King Abdullah University of Science and Technology (KAUST)Thuwal, 23955-6900, Saudi Arabia; 2 Department of Math and Statistics, Dalhousie UniversityHalifax, Nova Scotia, Canada, B3H 3J5; 3 Department of Biology, Dalhousie UniversityHalifax, Nova Scotia, Canada, B3H 3J5; 4 Department of Biology, The American University of CairoNew Cairo, 11835, Egypt

**Keywords:** Cyanophage, metagenomics, osmolyte, *Pelagibacter*, population genomics, *Prochlorococcus*, SAR11

## Abstract

Large swaths of the nutrient-poor surface ocean are dominated numerically by cyanobacteria (*Prochlorococcus*), cyanobacterial viruses (cyanophage), and alphaproteobacteria (SAR11). How these groups thrive in the diverse physicochemical environments of different oceanic regions remains poorly understood. Comparative metagenomics can reveal adaptive responses linked to ecosystem-specific selective pressures. The Red Sea is well-suited for studying adaptation of pelagic-microbes, with salinities, temperatures, and light levels at the extreme end for the surface ocean, and low nutrient concentrations, yet no metagenomic studies have been done there. The Red Sea (high salinity, high light, low N and P) compares favorably with the Mediterranean Sea (high salinity, low P), Sargasso Sea (low P), and North Pacific Subtropical Gyre (high light, low N). We quantified the relative abundance of genetic functions among *Prochlorococcus*, cyanophage, and SAR11 from these four regions. Gene frequencies indicate selection for phosphorus acquisition (Mediterranean/Sargasso), DNA repair and high-light responses (Red Sea/Pacific *Prochlorococcus*), and osmolyte C1 oxidation (Red Sea/Mediterranean SAR11). The unexpected connection between salinity-dependent osmolyte production and SAR11 C1 metabolism represents a potentially major coevolutionary adaptation and biogeochemical flux. Among *Prochlorococcus* and cyanophage, genes enriched in specific environments had ecotype distributions similar to nonenriched genes, suggesting that inter-ecotype gene transfer is not a major source of environment-specific adaptation. Clustering of metagenomes using gene frequencies shows similarities in populations (Red Sea with Pacific, Mediterranean with Sargasso) that belie their geographic distances. Taken together, the genetic functions enriched in specific environments indicate competitive strategies for maintaining carrying capacity in the face of physical stressors and low nutrient availability.

## Introduction

A handful of dominant microbial groups are found consistently in the tropical and subtropical surface ocean. Cyanobacteria of the genus *Prochlorococcus*, viruses (cyanophage) infecting *Prochlorococcus*, and proteobacteria of the SAR11 clade, together fill critical biogeochemical roles in primary production and cycling of carbon and nutrients. While these groups are ubiquitous, they are not homogenous. Populations in different seas and oceans exhibit phenotypes that reflect local environmental conditions, such as low nutrients, high salinity, or high irradiance. Community genomics (metagenomics) has become an important tool in marine microbial ecology, particularly in the comparison of multiple environments (comparative metagenomics) to reveal adaptive genotypes. Insights have included depth-dependent differences in taxonomic composition, gene functions, and metabolic potential (DeLong et al. 2006), spectral tuning of proteorhodopsins across the Atlantic, Pacific, and Indian Oceans (Rusch et al. 2007), and increased levels of phosphorus uptake genes in response to low phosphorus levels in the Sargasso Sea (Rusch et al. 2007; Coleman and Chisholm 2010; Martiny et al., 2011). The exploration of additional diverse environments, such as the unique and underexplored Red Sea, promises to reveal further adaptive mechanisms.

By most measures, the Red Sea lies at the extreme end of pelagic marine environments. Because of its low latitude and clear water, solar irradiance is high and penetrates deeply (Stambler 2005). The Red Sea is also among the most saline bodies of water in the world ocean (along with the Mediterranean Sea and Arabian Gulf), with surface salinity ranging from 36 to 41 psu (Edwards 1987). Temperatures regularly exceed 30°C at the surface in summer and fall and are isothermal (21–22°C) down to the ocean floor year-round (Edwards 1987). The Red Sea nevertheless resembles the major open-ocean gyres in that it is oligotrophic (Stambler 2005) and *Prochlorococcus* and SAR11 dominate its pelagic bacterioplankton (Lindell and Post 1995; Ngugi et al. 2012).

In the oligotrophic (nutrient-poor) surface waters that cover much of the tropical and subtropical ocean, *Prochlorococcus* (Chisholm et al. 1988), cyanophage (Bergh et al. 1989), and SAR11 (Giovannoni et al. 1990) are the dominant phototrophic, viral, and heterotrophic microbes, respectively. These groups occupy central roles in marine biogeochemistry. *Prochlorococcus* is a major contributor to oxygen evolution, carbon fixation, and primary production, in some places contributing half of all primary productivity (DuRand et al. 2001; Johnson 2006). Cyanophage (cyanobacterial viruses) are important predators of *Prochlorococcus* and its more coastal/eutrophic-adapted relative *Synechococcus* (Sullivan et al. 2003). These viruses contribute to host cyanobacterial mortality (Suttle and Chan 1994) and are important vectors for horizontal gene transfer among host cells (Coleman et al. 2006; McDaniel et al. 2010). The SAR11 clade (including ‘*Candidatus Pelagibacter* ubique’) is the most numerous group of marine organisms known (Rappé et al. 2002), playing important roles in nutrient cycling in the ocean. SAR11 obtains energy from both reduced carbon compounds and light energy (via proteorhodopsin) (Giovannoni et al. 2005a), yet much is still unknown about its metabolic capabilities. Grossly simplified, these three groups encapsulate much of the metabolic activity in the marine microbial loop: *Prochlorococcus* fixes carbon dioxide to sugar and biomass; cyanophage infect and lyse *Prochlorococcus*, releasing organic matter to the surrounding seawater; and SAR11 uses that organic matter to grow, in turn releasing as yet unrevealed metabolites back to *Prochlorococcus*. The recent discovery of widespread viruses (pelagiphage) that infect SAR11 (Zhao et al. 2013) heralds a new frontier in SAR11 gene transfer and biomass cycling with the community.

Comparative metagenomics is an effective tool for identifying functional differences in communities composed of dominant, well-studied taxonomic groups. Dominant taxa tend to constitute a large fraction of metagenomic reads, improving statistical power. Well-studied taxa will have reference genomes, characterized taxonomic subgroups, and physiological and biochemical knowledge, which all assist in the analysis process. For example, by building gene clusters from the sequenced genomes of *Prochlorococcus* and SAR11 and then assigning reads to those gene clusters, Coleman and Chisholm (2010) identified low phosphorus levels in the Sargasso Sea to be a major driving force in the adaptation of both taxonomic groups. The comparison of the Sargasso Sea and North Pacific Subtropical Gyre in that study provides a foundation for gene-centric studies of other pelagic marine microbial populations, namely, from the Mediterranean and Red Seas.

The Red Sea and Mediterranean contrast with the North Pacific and Sargasso Sea by being significantly more saline (Edwards 1987; Manca et al. 2004). Also, the deep-seawater masses of these two saline water bodies are isothermal and relatively warmer (22°C and ∼14°C, respectively) (Edwards 1987; Danovaro et al. 2010) than the average global ocean temperature, which decreases with depth to 3–5°C below 500 m. The Red Sea additionally experiences high annual solar irradiance (Edwards 1987). Although all four seas are considered oligotrophic—the Red Sea (Edwards 1987) and Mediterranean (Manca et al. 2004) more so in the interior northern and eastern parts of their respective basins—nutrient concentration ratios differ, considerably influencing growth of residing microbial communities. The Red Sea has moderately low N and P (Edwards 1987), the Mediterranean and Sargasso have very low P (Wu et al. 2000; Manca et al. 2004), and the North Pacific has very low N (Karl et al. 2001); because of isolation from dust sources, the North Pacific also has very low iron (Jickells et al. 2005). A comparative approach could reveal, for example, how *Prochlorococcus* deals with the high irradiance in the Red Sea, or how SAR11 copes with the high salinity in the Red Sea and Mediterranean.

In this study we asked, which microbial genetic functions are differentially represented in the Red Sea, how do these functions compare to those indicated in the Mediterranean, Sargasso, and North Pacific, and what do these functions tell us about adaption to and relationships among the four marine environments? Here we have analyzed the first microbial metagenome from the Red Sea, generated from a 50-m sample from an open-ocean site in the central Red Sea. Comparing these data to existing epipelagic metagenomes from the western Mediterranean (Ghai et al. 2010), the Bermuda Atlantic Time-series and the Hawaii Ocean Time-series (Coleman and Chisholm 2010), we were able to determine which genes are over- or under-represented in each of the four seas, and group both genes and seas according to gene distribution patterns. The results highlight both competition and cooperation in the survival strategies of oligotrophic marine microbes, and the functional variation responsible for these adaptions can be explained in part by the underlying phylogenetic variation. Finally, patterns of relatedness among different marine ecosystems hint at common adaptive mechanisms for surviving specific physicochemical stresses in geographically disparate seas.

## Methods

### Pyrosequenced microbial metagenomes

Seawater was collected from 50 m depth at the Atlantis II Deep area during the KAUST Red Sea Expedition in October 2008 (see Table 1 and Supporting Information for details about the samples and sampling locations). The small microbial size fraction containing *Prochlorococcus* and SAR11 (0.1–0.8 μm) was collected by filtration, DNA extracted, and pyrosequenced using a 454 GS FLX sequencer (Data S1). Existing pyrosequenced metagenomic datasets for surface microbial communities were obtained from previous studies of the Mediterranean deep chlorophyll maximum (Ghai et al. 2010), the Bermuda Atlantic Time-Series (BATS216), and the Hawaii Ocean Time-series (HOT186) (Coleman and Chisholm 2010).

**Table 1:** Database and source water properties for the four metagenomic datasets included in this study, including estimated nutrient concentrations and physical properties

	RS	MED	BATS			HOT		
				
	50 m	50 m	20 m	50 m	100 m	25 m	75 m	110 m
Database properties								
Sequence reads	1,177,603	1,204,381	357,881	464,651	525,605	623,558	673,673	473,165
Total base pairs (Mbp)	365	318	88	102	120	136	139	110
Mean read length (bp)	310	264	246	220	228	218	206	232
Median read length (bp)	327	273	263	247	251	249	223	254
Sample properties								
Site/cruise	Atlantis II	MedDCM	BATS216			HOT186		
Date collected	Oct. 2008	Oct. 2007	Oct. 2006			Oct. 2006		
Latitude	21.217°N	38.068°N	31.667°N			22.733°N		
Longitude	37.967°E	0.232°E	64.167°W			158.033°W		
Mixed layer depth (m)	45	n.a.	45			70		
Deep chl max depth (m)	80	50	100			110		
Size fraction (μm)	0.1–0.8	0.2–5.0	0.2–1.6			0.2–1.6		
Physicochemical properties								
Nitrate+Nitrite (μM)	0.21	0.50	<d.l.	<d.l.	0.12	0.06 ± 0.01	0.08	0.07
Nitrite (μM)	0.04	n.a.	0.01	0.01	0.02	n.a.	n.a.	n.a.
Phosphate (μM)	0.11	∼0.1	<d.l.	<d.l.	<d.l.	0.02 ± 0.01	0.02	0.05
Salinity (psu)	39.67 ± 0.01	∼38	36.44 ± 0.08	36.74 ± 0.08	36.69 ± 0.07	35.12 ± 0.05	35.20 ± 0.07	35.30 ± 0.01
Temperature (°C)	29.1 ± 0.2	∼16	26.7 ± 0.3	24.0 ± 1.2	19.6 ± 0.9	26.20 ± 0.05	23.5 ± 1.0	22.06 ± 0.06
Monthly mean solar downward flux (W m^−2^)								
Yearly mean	244.2	201.1	190.4			240.0		
Brightest month mean	307.5	315.0	285.2			309.4		
Dimmest month mean	173.6	89.4	94.1			157.3		

Where multiple data points were available, ranges of values (midpoint, minimum, maximum) are reported. See Methods for more information. Sequence read archive accession numbers for pyrosequencing reads: RS: SRX253027; MED: SRX017111; BATS: SRX008032, SRX008033, SRX008035; HOT: SRX007369, SRX007370, SRX007372. n.a., not available; d.l., detection limit.

### Physical and chemical parameters

Physical and chemical data for the four sampling sites were acquired directly or taken from literature and online databases (Table 1). Red Sea (RS) values are from the 2011 KAUST Red Sea Expedition, with nutrient analyses carried out at the UCSB Marine Science Institute. Mediterranean (MED) nutrient data are from June 1986 (Estrada et al. 1993), and salinity and temperature values are from World Ocean Atlas (http://www.nodc.noaa.gov/). BATS (http://bats.bios.edu/) and HOT (http://hahana.soest.hawaii.edu/) values are from respective cruises in October 2006. Water column conductivity, temperature, and density traces (Fig. S1) and additional information are in Supporting Information.

### Assignment of metagenomic reads to taxon-specific gene clusters

Coarse-scale taxonomic analysis of the metagenomes was first carried out on the 16S rRNA genes (see Supporting Information). Then, assignment of all metagenomic reads to taxonomic groups and gene clusters within those groups was done. Methods were similar to those of Coleman and Chisholm (2010) and are described fully in Supporting Information and Figure S2. Briefly, individual reads were assigned to taxonomic groups by comparison to GenBank-nr using BLASTX. Reads binned as *Prochlorococcus*, cyanophage, or SAR11 were then assigned to gene clusters using BLASTN against the respective sets of publicly available genomes. A read was assigned to a given gene cluster if the top three gene hits among the genomes belonged to the same gene cluster.

### Relative normalized gene cluster abundances across seas

Prior to normalization (Fig. S2B), gene clusters with total read counts of 20 or less across all samples were removed. Read counts for each gene cluster and sample were then normalized for each sample to the total number of recruited reads in all gene clusters. These normalized counts were then further normalized for each gene cluster across the samples. We call the resulting metric “relative normalized abundance” (r.n.a.). Shannon entropy was used to identify gene clusters with nonuniform abundance distributions. Using these calculated r.n.a. values and entropies, gene clusters were identified that were over- or under-represented in one of the samples. To be considered over- or under-represented, gene clusters were required to have an r.n.a. for that sample in the top or bottom 10% of gene clusters, an entropy in the lowest 15% (*Prochlorococcus* and SAR11) or 25% (cyanophage), and a total read count across all samples in the top 75%.

### Ecotype distributions of reads assigned to gene clusters

Relative contributions of different *Prochlorococcus* or cyanophage ecotypes (Table S1) to read counts for each gene cluster in each sea were calculated using the top BLASTN hits from above. For gene clusters designated as outliers by distance from the median (Fig. 2), an additional measure of outlierness was applied. Ecotype distributions were compared using Kullback–Leibler (KL) distances (Kullback and Leibler 1951), and those gene clusters with larger KL distances from the mean than 80% of the nonover-represented gene clusters were considered outliers.

### Clustering of seas by gene cluster abundance patterns

Hierarchical clustering was carried out using the program AGNES (Kaufman and Rousseeuw 2005) with KL distances (Kullback and Leibler 1951). To cluster the four seas, hierarchical clustering was performed on the normalized abundances for each sample, using only those gene clusters with entropy in the lowest 25% and a total read count across the four seas in the top 75%.

## Results

### Community composition of four marine metagenomes

The 16S rRNA profiles (Fig. S3) indicate that proteobacteria, especially SAR11 (Fig. S3B), and cyanobacteria, especially *Prochlorococcus* (Fig. S3C), are the most abundant microbial groups in the four metagenomes. Taxonomic profiling of all reads (Fig. S4) supports this distribution, showing that cyanophage also constitute a significant fraction of the total reads (metagenomic ‘bycatch’ of the filtration process). Deviations from the general trends include MED and BATS (20 m), which have less total cyanobacteria (Fig. S3A) but relatively more *Synechococcus* (Fig. S3C). In MED, a significant fraction of cyanobacteria are *Merismopedia* (Fig. S3C).

Classification of the metagenomic reads was further extended to subgroups or ecotypes within SAR11, *Prochlorococcus*, and cyanophage (Fig. S4). Only subgroups or ecotypes with sequenced genomes (Table S1) could be counted with this method. The distribution of assigned reads within the SAR11 populations in each sea is ∼89% subgroup 1a and ∼11% subgroup 3. The *Prochlorococcus* populations are dominated by the high-light II (HLII) clade, ∼80–95% in each sea except MED, where the high-light I (HLI) clade dominates. There is more *Prochlorococcus* from the low-light (LL) clades in BATS and HOT, as expected, because these datasets include samples from deeper waters than RS and MED. The cyanophage populations in each sea are dominated by T4-like cyanophage (>90%) relative to T7-like cyanophage (5–10%) and siphoviruses (<0.1%).

### Functional features of over-represented gene clusters among seas

**Figure 1:**
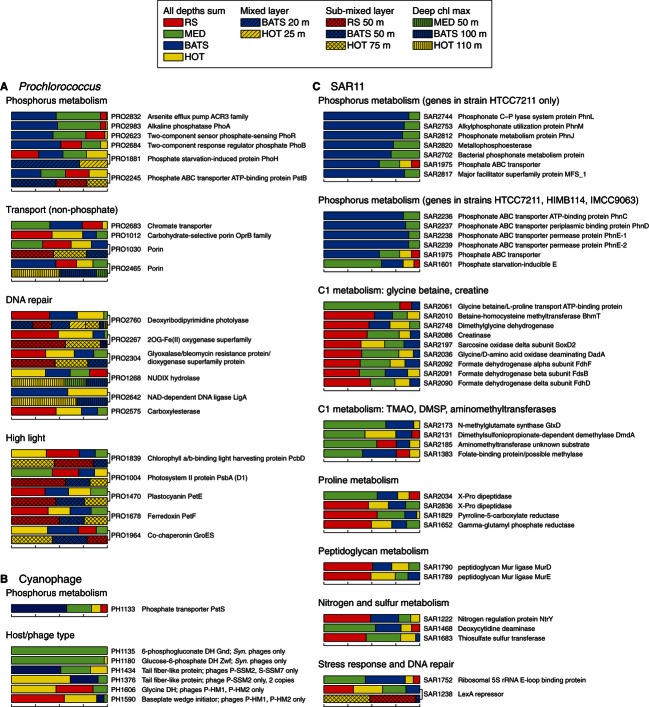
Stacked bar graphs showing relative normalized abundances of gene clusters over-represented in one or more of the four seas. Gene clusters implicated in selected metabolic processes are shown. Data shown are for all depths summed for each sea (solid colors), or for mixed layer depths only (diagonal shading), sub-mixed layer depths only (cross-hatched shading), or deep chlorophyll maximum depths only (horizontal shading). Bars are sorted by size from left (largest) to right (smallest). Tick marks indicate 25% subdivisions.

Relative normalized abundance (r.n.a.) calculations (Fig. S2) revealed gene clusters with low levels of Shannon entropy, that is, not evenly distributed and more likely to be found in one sea than another. Gene clusters over-represented in one or more of the four seas are listed (Table 2), and select gene clusters organized by functional category are plotted as bar graphs (Fig. 1). A list of gene clusters over- or under-represented in depth-specific comparisons is also provided (Table S3).

**Table 2:** Gene clusters over-represented in RS, MED, BATS, or HOT

	RS	MED	BATS	HOT	Entropy	Reads	Function	ProPortal	Distribution
*Prochlorococcus* genes over-represented in RS									
PRO2654	0.540	0.000	0.197	0.263	1.004	108	Hypothetical protein	3504	Core HLII
PRO2267	0.488	0.032	0.118	0.362	1.081	111	2OG-FeII oxygenase superfamily	4466	All except MED4
PRO2760	0.397	0.037	0.289	0.277	1.204	368	Deoxyribodipyrimidine photolyase	7370	4/5 HLII, 1/2 HLI
PRO2575	0.465	0.102	0.167	0.265	1.240	93	Carboxylesterase	3327	Core HL
PRO2420	0.445	0.077	0.246	0.231	1.242	122	MnII/FeII transporter	6754	Core HLII
PRO2498	0.363	0.057	0.309	0.271	1.248	119	LEM domain-containing protein	3045	Core HL
PRO1012	0.423	0.115	0.142	0.320	1.254	116	Carbohydrate-selective porin OprB family	4464	All except MED4
PRO2504	0.405	0.072	0.246	0.277	1.257	138	SMC domain-containing protein	3321	Core HL
*Prochlorococcus* genes over-represented in MED									
PRO2832	0.063	0.462	0.466	0.009	0.929	121	Arsenite efflux pump ACR3 family	3136	2/7 HL, 3/6 LL
PRO2983	0.055	0.449	0.480	0.016	0.937	325	Alkaline phosphatase PhoA	3127	2/7 HL, 2/6 LL
PRO2362	0.075	0.637	0.164	0.124	1.037	97	4-amino-4-deoxy-L-arabinose transferase	7522	Core HL
PRO2369	0.101	0.555	0.267	0.077	1.110	94	Hypothetical protein	3087	Core HLI, core LL
PRO2623	0.198	0.342	0.433	0.026	1.146	188	Two-component sensor kinase P-sensing PhoR	3125	3/7 HL, 4/6 LL
PRO2683	0.203	0.388	0.346	0.063	1.232	218	Chromate transporter	3130	3/7 HL, 3/6 LL
PRO3097	0.195	0.470	0.132	0.203	1.264	117	Peroxiredoxin DsrE family	2737	3/7 HL
*Prochlorococcus* genes over-represented in BATS									
PRO2832	0.063	0.462	0.466	0.009	0.929	121	Arsenite efflux pump ACR3 family	3136	2/7 HL, 3/6 LL
PRO2983	0.055	0.449	0.480	0.016	0.937	325	Alkaline phosphatase PhoA	3127	2/7 HL, 2/6 LL
PRO2524	0.287	0.000	0.406	0.308	1.087	119	Cytochrome c class I	4564	Core LL
PRO2623	0.198	0.342	0.433	0.026	1.146	188	Two-component sensor kinase P-sensing PhoR	3125	3/7 HL, 4/6 LL
PRO2684	0.215	0.200	0.515	0.070	1.181	122	Two-component response regulator P PhoB	3124	3/7 HL, 3/6 LL
PRO2683	0.203	0.388	0.346	0.063	1.232	218	Chromate transporter	3130	3/7 HL, 3/6 LL
PRO2216	0.313	0.064	0.347	0.276	1.262	163	Rhodanese-like protein	2514	All except MIT9202
*Prochlorococcus* genes over-represented in HOT									
PRO2267	0.488	0.032	0.118	0.362	1.081	111	2OG-FeII oxygenase superfamily	4466	All except MED4
PRO1312	0.310	0.043	0.319	0.328	1.228	258	Abortive infection protein	2716	Core
PRO2365	0.308	0.048	0.314	0.330	1.239	151	Hypothetical protein	5598	5/7 HL, core LL
Cyanophage genes over-represented in RS									
PH1590	0.551	0.034	0.076	0.340	1.004	100	Baseplate wedge initiator	93	P-HM1, P-HM2 only
PH1063	0.526	0.282	0.000	0.192	1.012	40	Plasmid stability protein	166	All T4-like except S-PM2
PH1210	0.599	0.056	0.255	0.090	1.034	75	Hypothetical protein	108	5/17 T4-like
PH1309	0.435	0.328	0.000	0.236	1.069	114	Hypothetical protein	373	3/17 T4-like
Cyanophage genes over-represented in MED									
PH1105	0.000	1.000	0.000	0.000	0.000	40	Hypothetical cyanophage protein	258	Syn T4-like only (10/17)
PH1135	0.000	1.000	0.000	0.000	0.000	54	6-phosphogluconate dehydrogenase Gnd	964	Syn T4-like only (8/17)
PH1180	0.000	0.968	0.000	0.032	0.142	38	Glucose-6-phosphate dehydrogenase Zwf	969	Syn T4-like only (6/17)
PH1046	0.095	0.519	0.000	0.386	0.931	52	Terminase DNA packaging enzyme small subunit	106	Core T4-like
PH1144	0.192	0.469	0.000	0.339	1.039	42	Precursor of major head subunit	1074	8/17 T4-like
PH1009	0.365	0.445	0.000	0.190	1.043	46	Hypothetical protein	233	Core T4-like
Cyanophage genes over-represented in BATS									
PH1168	0.016	0.359	0.607	0.018	0.807	37	DUF680 domain-containing protein	173	7/17 T4-like
PH1434	0.000	0.310	0.515	0.175	1.010	44	Phage tail fiber-like protein	93	P-SSM2, S-SSM7 only
PH1133	0.068	0.259	0.577	0.096	1.076	223	Phosphate transporter PstS	174	9/17 T4-like
Cyanophage genes over-represented in HOT									
PH1145	0.241	0.084	0.000	0.675	0.816	40	Hypothetical protein	336	8/17 T4-like
PH1574	0.393	0.047	0.000	0.560	0.835	37	Hypothetical protein	2051	P-HM1, P-HM2 only
PH1376	0.000	0.096	0.290	0.614	0.884	37	Phage tail fiber-like protein	564	P-SSM2 only (2 copies)
PH1606	0.387	0.148	0.000	0.465	1.006	169	Glycine dehydrogenase	2105	P-HM1, P-HM2 only
PH1158	0.309	0.212	0.000	0.479	1.044	38	Hypothetical protein	1048	7/17 T4-like
PH1033	0.244	0.337	0.000	0.419	1.075	103	Recombination endonuclease subunit	138	Core T4-like

	RS	MED	BATS	HOT	Entropy	Reads	Function	Distribution
SAR11 genes over-represented in RS								
SAR1829	0.557	0.288	0.124	0.031	1.051	47	Pyrroline-5-carboxylate reductase	Core
SAR1935	0.506	0.335	0.091	0.068	1.113	43	Hypothetical protein PU1002_05631	All except HTCC7211
SAR1683	0.441	0.360	0.042	0.158	1.153	46	Probable thiosulfate sulfur transferase	Core
SAR1222	0.489	0.124	0.291	0.095	1.192	73	Nitrogen regulation protein NtrY	Core
SAR2036	0.394	0.386	0.067	0.152	1.202	58	Glycine/D-amino acid oxidase deaminating DadA	Core SG1a
SAR2086	0.451	0.334	0.099	0.116	1.204	88	Creatinase	Core SG1a
SAR2010	0.526	0.154	0.188	0.131	1.207	63	Betaine-homocysteine methyltransferase BhmT	Core SG1a
SAR1136	0.503	0.229	0.107	0.162	1.217	43	Rhodanese domain protein	Core
SAR1790	0.510	0.114	0.198	0.179	1.219	45	Peptidoglycan Mur ligase MurD	Core
SAR1023	0.474	0.142	0.101	0.283	1.220	60	Short chain dehydrogenase	All except HIMB114
SAR1789	0.491	0.133	0.122	0.255	1.222	129	Peptidoglycan Mur ligase MurE	Core
SAR1788	0.438	0.141	0.101	0.320	1.234	85	Cell division protein FtsI	Core
SAR1652	0.496	0.137	0.154	0.213	1.237	69	Gamma-glutamyl phosphate reductase	Core
SAR11 genes over-represented in MED								
SAR2061	0.123	0.793	0.084	0.000	0.650	55	Glycine betaine/L-proline transporter ATP-binding	Core SG1a
SAR1835	0.090	0.652	0.026	0.232	0.929	41	Hypothetical protein PU1002_01756	HTCC1002, 1062
SAR1945	0.184	0.658	0.052	0.105	0.978	62	Type II secretion system protein PilY1	All except HIMB114
SAR1601	0.059	0.600	0.227	0.114	1.058	43	Phosphate starvation-inducible E	Core
SAR2062	0.217	0.581	0.153	0.049	1.081	134	Hypothetical protein PU1002_02421	Core SG1a
SAR2725	0.106	0.611	0.145	0.137	1.092	59	Trap dicarboxylate transporter dctm subunit	HTCC7211 only
SAR1752	0.066	0.433	0.415	0.085	1.117	56	Ribosomal 5S rRNA E-loop binding protein	Core
SAR1468	0.085	0.538	0.267	0.110	1.139	44	Deoxycytidine triphosphate deaminase	Core
SAR2441	0.044	0.434	0.379	0.143	1.146	55	ABC sugar transporter	HTCC1062, 7211
SAR2034	0.109	0.557	0.213	0.120	1.152	80	X-Pro dipeptidase	Core SG1a
SAR1817	0.146	0.562	0.166	0.125	1.163	51	Phosphoglycerate dehydrogenase	Core
SAR2185	0.204	0.552	0.139	0.105	1.163	91	Aminomethyltransferase unknown substrate	Core SG1a
SAR2319	0.075	0.411	0.398	0.116	1.177	81	Putative ABC transport protein	HTCC1062, 7211
SAR1814	0.171	0.495	0.271	0.063	1.177	51	Penicillin binding protein transpeptide	Core
SAR1463	0.331	0.463	0.109	0.098	1.191	47	ATP phosphoribosyltransferase	Core
SAR1306	0.102	0.483	0.284	0.131	1.209	48	ABC transporter	Core
SAR2787	0.169	0.516	0.126	0.190	1.218	57	Ectoine/hydroxyectoine ABC transporter solute-binding	HTCC7211 only
SAR1461	0.260	0.406	0.051	0.283	1.226	56	Thioredoxin 1	Core
SAR2124	0.277	0.470	0.152	0.102	1.229	61	Glutamine amidotransferase class I	Core SG1a
SAR2131	0.083	0.428	0.166	0.322	1.234	41	Dimethylsulfoniopropionate-dependent demethylase DmdA	Core SG1a
SAR1373	0.277	0.422	0.236	0.065	1.238	48	Outer membrane protein TolC	Core
SAR11 genes over-represented in BATS								
SAR2744	0.000	0.112	0.888	0.000	0.351	45	Phosphonate C-P lyase system protein PhnL	HTCC7211 only
SAR2238	0.000	0.113	0.887	0.000	0.353	52	Phosphonate ABC transporter permease protein PhnE-1	HTCC7211, core SG3
SAR2239	0.000	0.115	0.885	0.000	0.358	102	Phosphonate ABC transporter permease protein PhnE-2	HTCC7211, core SG3
SAR2237	0.000	0.146	0.854	0.000	0.417	52	Phosphonate ABC transporter periplasmic-binding PhnD	HTCC7211, core SG3
SAR2817	0.000	0.159	0.841	0.000	0.438	64	Major facilitator superfamily MFS_1 putative	HTCC7211 only
SAR2753	0.000	0.161	0.839	0.000	0.442	79	Alkylphosphonate utilization protein PhnM	HTCC7211 only
SAR2236	0.000	0.170	0.830	0.000	0.456	60	Phosphonate ABC transporter ATP-binding protein PhnC	HTCC7211, core SG3
SAR2812	0.000	0.179	0.821	0.000	0.470	57	Phosphonate metabolism protein PhnJ	HTCC7211 only
SAR2792	0.000	0.215	0.785	0.000	0.520	48	Diacylglycerol kinase catalytic domain protein	HTCC7211 only
SAR2820	0.000	0.274	0.726	0.000	0.587	54	Metallophosphoesterase	HTCC7211 only
SAR2702	0.000	0.298	0.702	0.000	0.609	41	Bacterial phosphonate metabolism protein	HTCC7211 only
SAR2815	0.113	0.237	0.650	0.000	0.867	43	Glycosyl transferase group 1	HTCC7211 only
SAR3069	0.000	0.213	0.635	0.152	0.904	69	SnoK-like protein	IMCC9063 only
SAR1783	0.071	0.249	0.649	0.031	0.922	51	Lipoprotein precursor	Core
SAR3070	0.016	0.202	0.534	0.248	1.070	70	Hypothetical protein	IMCC9063 only
SAR2709	0.061	0.214	0.593	0.131	1.078	57	20S proteasome A/B subunit	HTCC7211 only
SAR1975	0.086	0.211	0.579	0.125	1.115	108	Phosphate ABC transporter	All except HTCC1002
SAR1752	0.066	0.433	0.415	0.085	1.117	56	Ribosomal 5S rRNA E-loop binding protein	Core
SAR1784	0.140	0.177	0.575	0.108	1.141	41	Probable N-acetylmuramoyl-L-alanine amidase	Core
SAR2441	0.044	0.434	0.379	0.143	1.146	55	ABC sugar transporter	HTCC1062, 7211
SAR2717	0.066	0.298	0.499	0.137	1.160	89	Hypothetical protein	HTCC7211 only
SAR2319	0.075	0.411	0.398	0.116	1.177	81	Putative ABC transport protein	HTCC1062, 7211
SAR1002	0.340	0.238	0.386	0.036	1.196	41	Fumarylacetoacetate hydrolase family protein	Core
SAR1863	0.107	0.376	0.413	0.104	1.208	45	ABC-type sugar transport system	Core SG1a
SAR1904	0.159	0.086	0.482	0.272	1.210	60	Histone deacetylase family protein	All except IMCC9063
SAR1131	0.094	0.330	0.423	0.152	1.239	50	Putative Holliday junction resolvase	Core
SAR11 genes over-represented in HOT								
SAR1341	0.174	0.295	0.021	0.510	1.089	41	Ribosomal protein S6	Core
SAR2783	0.171	0.099	0.169	0.560	1.157	387	Cell wall-associated hydrolase	HTCC7211 only

For each gene cluster, relative normalized abundance in each of the four seas, entropy, number of reads mapping, proposed function, cross-referenced ProPortal CyCOG (*Prochlorococcus*) and PhCOG (cyanophage) numbers (http://proportal.mit.edu/), and distribution among the genomes are given. Data for BATS and HOT were summed over three depths (Methods). Genome information for distributions can be found in Table S1.

*Prochlorococcus* gene clusters differentially represented in the data (Table 2 and Fig. 1A) fall into two major categories: nutrient stress and acquisition, especially phosphorus; and high-light/UV stress, including DNA repair pathways. BATS and MED are both enriched in genes for phosphorus acquisition, including alkaline phosphatase, phosphate-sensing two-component system PhoBR, and an arsenite efflux pump. The BATS mixed layer sample is enriched in the putative phosphate-related protein PhoH, and the BATS sub-mixed layer is enriched in the phosphate transporter PstB (Fig. 1A and Table S3). Other transporters, including a chromate transporter and several porins, are over-represented in different samples. RS and HOT are both enriched in genes involved in DNA repair and light stress (Fig. 1A). The DNA repair genes include 2-oxoglutarate–Fe(II) oxygenase, deoxyribopyrimidine photolyase, NAD-dependent DNA ligase, and NUDIX hydrolase. The light stress-related genes include chlorophyll a/b-binding light-harvesting protein PcbD, photosystem II protein PsbA, plastocyanin, and ferredoxin.

Cyanophage gene clusters over-represented in certain samples (Table 2 and Fig. 1B) tend to be linked to host and phage type. That is, they represent genes restricted either to phages infecting only certain hosts (e.g., *Synechococcus* phages only) or to a small number of closely related phages (e.g., P-HM1 and P-HM2). The one notable exception is the host-like phosphate transporter PstS, which is over-represented in BATS and MED.

SAR11 gene clusters with over-representation in one or more samples (Table 2 and Fig. 1C) show two major trends: phosphorus acquisition, especially from phosphonates; and one-carbon (C1) metabolism, especially involving degradation of osmolytes. Phosphonate and phosphate acquisition genes are especially over-represented in BATS and to a lesser extent MED; in RS and HOT, phosphate-related genes are found but at low levels, and phosphonate-related genes are absent. Among the sequenced SAR11 genomes, the over-represented P-related genes are found either only in strain HTCC7211 (e.g., phosphonate C–P lyase) or only in strains HTCC7211, HIMB114, and IMCC9063 (e.g., phosphonate ABC transporter) (Fig. 1C). Osmolyte demethylation and C1 metabolism genes are over-represented in RS and MED. RS is enriched in genes for all the enzymes to convert glycine betaine (GBT) and creatine to glycine, plus formate dehydrogenase for the terminal oxidation of formate (Sun et al. 2011). MED is enriched in steps for utilizing trimethylamine N-oxide (TMAO) and dimethylsulfoniopropionate (DMSP) via the C1 degradation pathway (Sun et al. 2011), and also contains several aminomethyltransferases (AMTs). Additional genes for proline metabolism, peptidoglycan synthesis, nitrogen and sulfur metabolism, and stress response are also over-represented in RS and MED.

### Ecotype distributions of gene clusters of *Prochlorococcus* and cyanophage

**Figure 2:**
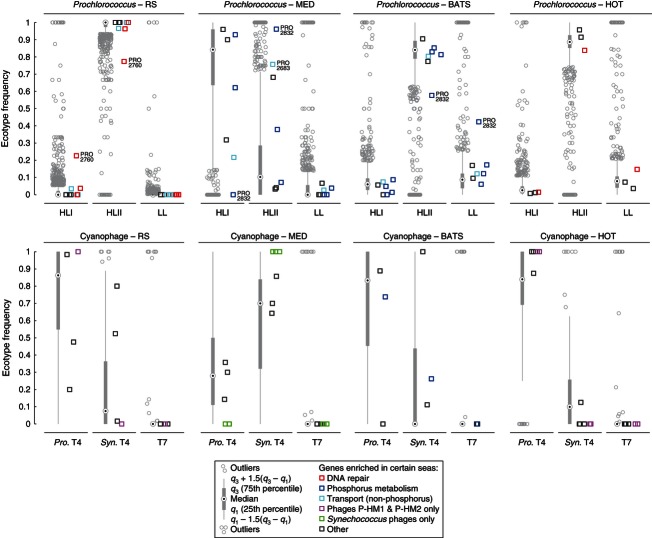
Ecotype distributions of gene clusters from *Prochlorococcus* and cyanophage. For each sea, ecotype frequencies for all gene clusters are plotted as box and whisker plots, with median, interquartile range, whiskers (whisker length *w* = 1.5), and outliers (outside of whiskers as defined) indicated. Colored boxes to the right of the box plots are gene clusters over-represented in that sea (Table 2), colored by metabolic function or phage distribution, with those gene clusters among the outliers labeled with the gene cluster number.

Relative contributions of different *Prochlorococcus* ecotypes and cyanophage types to read counts for each gene cluster in each sea were assessed (Fig. 2). The analysis was confined to *Prochlorococcus* and cyanophage because there is not yet a reliable ecotype paradigm for SAR11. The results echo the total-read ecotype distributions (Fig. S4), but here T4-like cyanophage are subdivided by host of isolation. Among T4-like cyanophage in RS, BATS, and HOT, *Prochlorococcus* T4-like phage predominate, while in MED, *Synechococcus* T4-like phage predominate.

Ecotype distributions are similar between gene clusters enriched in one of the four seas and the group of all gene clusters (Fig. 2). Exceptions are PRO2760 (photolyase) in RS *Prochlorococcus*, which has more HLI reads and fewer HLII reads than most gene clusters; PRO2832 (arsenite efflux pump) and PRO2683 (chromate transporter) in MED *Prochlorococcus*, which have more HLII reads and fewer HLI reads than most gene clusters; and PRO2832 (arsenite efflux pump) in BATS *Prochlorococcus*, which has more LL reads and fewer HLII reads than most gene clusters.

### Genomic context of gene cluster abundances among seas

**Figure 3:**
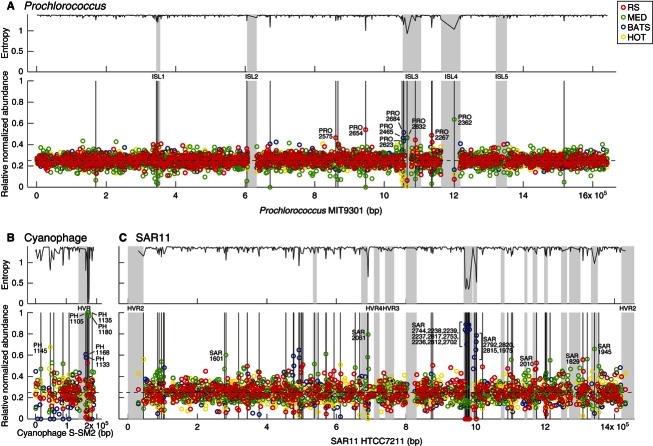
Relative normalized abundance and entropy of gene clusters versus position in reference genomes. Gene clusters are plotted at their corresponding positions in the reference genomes *Prochlorococcus* MIT9301, cyanophage S-SM2, and SAR11 HTCC7211, which were the most represented genomes based on top BLASTX hits (Methods). Only gene clusters with hit counts in the top 75% across the four seas are shown. Solid black lines indicate gene clusters with entropy in the bottom 15% (*Prochlorococcus*, SAR11) or 25% (cyanophage) and r.n.a. for one sea in the top or bottom 10%. Gray boxes indicate HVRs (Supporting Information). Dashed lines indicate equal normalized abundance across the four seas.

Relative normalized abundance (r.n.a.) of gene clusters from *Prochlorococcus*, cyanophage, and SAR11 was plotted as a function of position in highly represented reference genomes (Fig. 3). This approach reveals stretches of genomes that are collectively over-represented in certain environments, and it can identify possible hot spots of genetic recombination.

The most represented *Prochlorococcus* genome in the datasets, strain MIT9301, has several distinct regions with skewed abundances (Fig. 3A). Many of these regions correspond to known hypervariable regions (HVRs) in high light-adapted (HL) *Prochlorococcus* genomes. Coleman et al. (2006) defined five HVRs or genomic islands (ISL1–5) in HL *Prochlorococcus*, which show different levels of variability across the metagenomes. ISL1 and ISL3 are moderately variable and contain a significant fraction of the ecosystem-specific gene clusters. ISL1 contains a string of gene clusters enriched in RS and depleted in MED, most of which are annotated only as conserved hypothetical proteins. ISL3 contains a number of phosphate-related gene clusters that are enriched in MED and BATS, depleted in HOT, and present at low levels in RS. ISL2 and ISL4 are highly variable, almost entirely lacking any representation (i.e., not enough metagenomic reads could be recruited to calculate an r.n.a.). ISL4 contains viral attachment genes, and variability in ISL4 has been shown to be a major host defense against phage infection (Avrani et al. 2011). ISL5 has relatively low variability, with little in the data to distinguish it from the rest of the genome.

Diversity across cyanophage (Fig. 3B) and SAR11 (Fig. 3C) genomes was much greater than in *Prochlorococcus*. Phage genomes are known to be highly variable and mosaic in nature (Hendrix et al. 2000), and there is likewise broad variability among the SAR11 clade (Wilhelm et al. 2007). Both groups exhibited high variability in r.n.a. values and low entropy along the reference genomes, some of it localized to HVRs. In cyanophage S-SM2, the most differentially represented gene clusters—three genes specific for *Synechococcus* T4-like cyanophages and found almost exclusively in MED—occur in a previously identified HVR in T4-like cyanophages (Millard et al. 2009; Sullivan et al. 2010). In SAR11 HTCC7211, we identified a large number of HVRs (Table S2), three of which correlate to previously identified HVRs in SAR11 HTCC1062 (Wilhelm et al. 2007). Interestingly, most of the metagenomic gene cluster diversity is not found in these HVRs but rather in previously unidentified HVRs or outside of identified HVRs altogether. One of the newly identified HVRs, located around 10 × 10^5^ bp (Fig. 3C and Table S2), contains numerous genes for phosphonate utilization, a feature identified by Coleman and Chisholm (2010). Finally, in all three taxonomic groups, non-single-copy gene clusters were more likely to be differentially represented (Fig. S5) and have low entropies (Fig. S6) than single-copy gene clusters.

### Patterns of relatedness among seas based on gene cluster abundances

**Figure 4:**
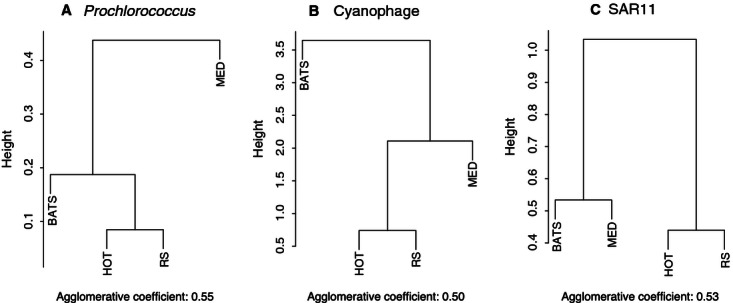
Hierarchical clustering of microbial populations from RS, MED, BATS, and HOT based on relative normalized abundances of gene clusters. Separate clustering patterns are shown for *Prochlorococcus*, cyanophage, and SAR11. The AGNES agglomerative coefficient measures separation between clusters, ranging from 0 (no structure found) to 1 (clear structure found) (Kaufman and Rousseeuw 2005). Resulting values range from 0.50 to 0.55 for the three groups, indicating that moderately clear structuring is detected.

To find patterns of genomic relatedness among the microbial populations, we clustered the four seas based on their gene cluster abundance values. Hierarchical clustering was carried out for each of the three major taxonomic groups (Fig. 4). Similar patterns were observed in all three taxonomic groups: RS clusters with HOT for each of the three groups. The distance of MED and BATS from the RS/HOT cluster is somewhat different for each taxonomic group. For *Prochlorococcus*, MED is the most distant; for cyanophage, BATS is the most distant; and for SAR11, MED and BATS are equally distant and clustered together.

## Discussion

### Comparative metagenomics of four seas

The goal of this study was to identify ecosystem-specific adaptations in marine microbial communities as revealed through the relative abundance of genomic potentials, with a special focus on the Red Sea. To achieve this goal, we have built upon previous studies, for example, using established methods for assigning metagenomic reads to gene clusters (Coleman and Chisholm 2010). At the same time, we have employed statistical tools new to metagenomics, such as the concepts of Shannon entropy and r.n.a., to help us discover differentially represented gene clusters among more than two datasets. We note that the gene clusters used to recruit metagenomic reads were generated from existing genomes only. We have done this to increase the certainty with which reads can be assigned taxonomically and to particular genetic functions, but we acknowledge that novel genes in the datasets are left out. Efforts focused on both metagenomic reads (e.g., assembly) and reference genomes (e.g., single-cell genomes and more genomes of isolates) will help glean more information from marine metagenomes in the future.

### Community composition

Phylogenetic classification of the four metagenomes (Fig. S4) provides some initial hints at ecological specialization. The Mediterranean site is more eutrophic and cooler than the other three sites (Table 1), and the metagenomic data reflect this: MED has four times more *Synechococcus* reads than the other datasets and one-fourth to one-half as many *Prochlorococcus* reads, which are predominantly HLI clade rather than HLII as in the other three sites. Indeed, *Synechococcus* is known to thrive in more nutrient-rich waters, and the preference of HLI for lower temperatures than HLII is documented (Johnson 2006). The high proportion of *Merismopedia* cyanobacteria in MED is also consistent with its more eutrophic status (Ghai et al. 2012). Previous studies support our findings regarding *Prochlorococcus* ecotype distribution in the western Mediterranean: Analysis of the 1999 PROSOPE expedition shows the western/central Mediterranean to be dominated by HLI *Prochlorococcus* at the surface (Garczarek et al. 2007). Interestingly, this contrasts with the eastern Mediterranean, which was shown to be predominantly HLII *Prochlorococcus* in surface waters along a transect from Israel to Cyprus (Feingersch et al. 2010). The source of this east–west ecotype difference remains unknown. The eastern Mediterranean is significantly more oligotrophic and P-limited than the western Mediterranean (Krom et al. 1991). Although the main environmental factor correlated with the relative abundance of HLI versus HLII is temperature (Johnson 2006), it may be that HLII *Prochlorococcus* are better adapted to oligotrophic or low-P conditions than HLI *Prochlorococcus*. It has also been proposed that the Red Sea may have inoculated the Mediterranean with HLII *Prochlorococcus* via the Suez Canal (Feingersch et al. 2010).

Several other patterns emerge in the community composition data, which require specific cell and virus counts to substantiate. HOT appears somewhat distinct from the other datasets, as it has significantly fewer SAR11 and more *Prochlorococcus* sequences. The data also seem to suggest that RS is enriched in cyanophage, with twice as many sequences as the next highest datasets (MED and HOT), but this may result from the smaller pore size of the filters used in the Red Sea (0.1 μm lower limit vs. 0.2 μm in other seas; Table 1).

We now consider ecosystem-specific adaptations in the three major groups individually, as indicated by the relative normalized abundances of gene clusters. We then discuss the greater implications of these adaptations for microbial ecology of the oceans.

### Ecosystem-specific adaptations in *Prochlorococcus*

As a photoautotroph dependent on sunlight for growth, *Prochlorococcus* is especially vulnerable to UV-induced DNA damage, photoinhibition of photosystem II, and reactive oxygen species (ROS) generated from overwhelmed photosynthetic electron transport (Scanlan et al. 2009). To deal with these solar insults, *Prochlorococcus* has various DNA repair pathways, photosystem repair mechanisms, and membrane protection pathways. Over-representation of genes for DNA repair and light stress in RS and HOT is likely an adaptation to the high irradiances experienced in these seas (Table 1), which are a function of low latitude, less annual cloud cover, and diminished particulate matter in the water column (Dishon et al. 2012). Because light is attenuated with depth, some of the light-related gene clusters are differentially represented depending on sample depth (Fig. 1A). Among the DNA repair genes, photolyase (for repairing pyrimidine dimers), which we found in the mixed and sub-mixed layers in RS, HOT, and BATS, has been previously been found at high levels in surface seawater metagenomes (DeLong et al. 2006; Singh et al. 2009) and metatranscriptomes (Frias-Lopez et al. 2008). Nucleic acid damage by alkylation can be repaired by 2-oxoglutarate–Fe(II) oxygenases (Falnes et al. 2002), and similar enzymes encoded by cyanophage genomes have a proposed role in DNA repair (Weigele et al. 2007; Sullivan et al. 2010); although the exact function of the enzyme highly enriched in RS and HOT remains unknown, it appears to have heightened importance for the high-irradiance at these sites. Photosystem II protein PsbA, known to turn over rapidly in high light (Kulkarni and Golden 1994), was over-represented in the sub-mixed layer in RS. RS also has higher levels of plastocyanin and ferredoxin genes, which encode electron carriers that maintain electron flow to prevent ROS formation under high light (Latifi et al. 2009).

The requirement of *Prochlorococcus* for inorganic nutrients—especially phosphorus, nitrogen, and iron—presents an additional challenge in the oligotrophic ocean. Nutrient limitation can be particularly acute in the low-phosphorus (high N/P ratio) waters of the Mediterranean and Sargasso Seas (Table 1). The over-representation of both *Prochlorococcus* and SAR11 phosphorus-related genes in the Sargasso Sea and their likely selective advantage was the subject of several recent studies (Sowell et al. 2008; Coleman and Chisholm 2010). Similarly, a survey of the eastern Mediterranean found enriched levels of phosphorus utilization genes, but they were assigned mostly to SAR11 and other alphaproteobacteria (Feingersch et al. 2010). Here we report that the western Mediterranean (MED) *Prochlorococcus* population, like that at BATS, has enriched genes for several mechanisms for dealing with low environmental phosphorus levels: Transcriptional activation of genes in response to low phosphate (PhoBR), harvesting of organic phosphate (PhoA), and arsenite efflux following nonselective uptake of arsenate with phosphate (ACR3) (Sanders and Windom 1980). Many of the phosphorus-related genes are found in HVRs of the *Prochlorococcus* genome (ISL3, Fig. 3A), corroborating previous reports (Coleman and Chisholm 2010). Notably, adaptations to low phosphorus are not confined to MED and BATS; RS also shows elevated levels of phosphorus acquisition genes relative to HOT (Fig. 1A).

In addition to inorganic and organic phosphate, phosphonate (organic) and phosphite (inorganic) are ready sources of phosphorus in the surface ocean, and *Prochlorococcus* has evolved to utilize them. Genomic sequencing (Kettler et al. 2007; Martinez et al. 2010) and functional screens (Martinez et al. 2010) indicate the capacity for *Prochlorococcus* to use phosphite and phosphonates. Calorimetry shows the *Prochlorococcus* transporters to have high affinities for phosphite or phosphonates (Feingersch et al. 2012). Further, certain *Prochlorococcus* strains are able to incorporate phosphite in culture (Martinez et al. 2012). We have not remarked thus far on these potentially significant environmental sources of phosphorus because they were not indicated in the data. One might expect over-representation of phosphite and phosphonate utilization genes in the Mediterranean and Sargasso Seas. Indeed, the putative phosphite and phosphonate transporters from *Prochlorococcus* are expressed (mRNA) in at sites in the Atlantic Ocean sites but not in the Pacific Ocean (Feingersch et al. 2012). However, the two three-gene cassettes found in *Prochlorococcus* (*phnDCE*, found in all *Prochlorococcus*, putatively phosphite-specific; *phnCDE*, strains MIT9301 and MIT9303 only, putatively phosphonate-specific) failed to surpass our assigned thresholds (r.n.a. in top 10% of gene clusters, entropy in lowest 15%, total read count in top 75%). The data indicate that *phnDCE* was evenly distributed across the four metagenomes, which is consistent with its being found once in each genome (i.e., core and single-copy). The metatranscriptome data cited in Feingersch et al. (2012) suggest that regulated expression of these genes in low-phosphorus environments determines their use by the population rather than gene presence/absence. *phnCDE*, the possible phosphonate acquisition system found in only two sequenced genomes, was indeed differentially represented: it was heavily enriched in MED and BATS, less abundant in RS, and virtually absent from HOT. However, total read counts of *phnCDE* were very low, indicating that while this gene cassette is relatively important in low-phosphorus waters, it has likely not swept through the entire *Prochlorococcus* population in these environments.

### Ecosystem-specific adaptations in cyanophage

Cyanophage are predominantly lytic viruses, infecting *Prochlorococcus* or *Synechococcus* and using host biomass and energy to reproduce. Cyanophage are therefore limited by many of the same factors as their hosts, such as high light or oxidative stress, carbon availability, or nutrients like phosphorus. Cyanophage have evolved to deal with these limitations by acquiring genes for various host metabolic processes, called ‘auxiliary metabolic genes’ (Thompson et al. 2011). The most notable over-represented cyanophage gene cluster is the phosphate transporter PstS in BATS and MED (Fig. 1B). PstS is expressed in cyanophage via exploitation of the host's phosphate-sensing mechanism (Zeng and Chisholm 2012). High frequencies of PstS in BATS and MED signify that not only bacteria but also viruses experience the selective pressure of low phosphorus levels. Genes for pentose phosphate pathway enzymes 6-phosphogluconate dehydrogenase and glucose-6-phosphate dehydrogenase are abundant in MED, as seen in the spike at the 3′-end of the S-SM2 genome (Fig. 3B). However, their over-representation in MED is likely due not to any special importance of the pentose phosphate pathway in the Mediterranean, but rather to there being significantly more *Synechococcus* in the Mediterranean (Fig. S3B) and only *Synechococcus* T4-like cyanophages carrying these two genes (Thompson et al. 2011).

### Ecosystem-specific adaptations in SAR11

As a chemoheterotroph, SAR11 requires organic carbon for energy and growth in addition to inorganic nutrients like phosphorus, sulfur, and nitrogen (Giovannoni et al. 2005b; Tripp et al. 2009). Incubation studies indicate that SAR11 can obtain organic carbon from amino acids and glucose (Malmstrom et al. 2005), yet media enrichments (Tripp et al. 2009) and genomic evidence (Giovannoni et al. 2005b) suggest that osmolytes may also be a major source of both energy and nutrients like nitrogen and sulfur. Osmolytes are used by many marine bacteria for osmotic regulation in saline environments (Burg and Ferraris 2008). Radiolabeling of C1 compounds has confirmed that osmolytes GBT, TMAO, and DMSP are demethylated and oxidized by SAR11 in culture (Sun et al. 2011). Transporters for these compounds and another osmolyte, proline, are encoded in SAR11 genomes (Giovannoni et al. 2005b). If osmolytes are excreted or released by lysis to the surrounding seawater, everything else being equal, they should be present in greater concentrations in saltier environments like the Red Sea and Mediterranean (Table 1). Indeed, the increased frequencies of gene clusters for degrading GBT, TMAO, DMSP, and proline in SAR11 from MED and especially RS (Fig. 1C) may indicate adaptation to increased osmolyte concentrations in those seas. These osmolytes could supply energy as well as sulfur and nitrogen, especially in nitrogen-limited environments like the Red Sea (Post 2005).

Regarding phosphorus, the over-representation in BATS and MED of phosphorus-related genes in SAR11 mirrors what we found in *Prochlorococcus* and cyanophage. As was observed in the original study of the BATS dataset (Coleman and Chisholm 2010) and a BAC end-sequence library from the eastern Mediterranean (Feingersch et al. 2010), we observed significant over-representation of SAR11 genes for phosphate and phosphonate utilization at these two sites. Unlike the case with *Prochlorococcus*, the majority of enriched phosphorus-related genes were for phosphonate specifically (Fig. 1C). If *Prochlorococcus* has a limited ability (relative to SAR11) to utilize phosphonates, this is ameliorated in part by its use of sulfolipids in place of phospholipids (Van Mooy et al. 2009), which reduces its phosphorus quota and minimizes competition for phosphorus with groups like SAR11.

### Salinity, osmolyte production, and SAR11 catabolism

The link between salinity, osmolytes, and C1 metabolism in SAR11 has significant implications for marine biogeochemistry. We expected to find direct adaptations for coping with high salinity, but what we found instead was a secondary effect: SAR11 (putatively) consumes the osmolytes produced by *Prochlorococcus* and other phytoplankton to cope with high salinity. *Prochlorococcus* is known to produce osmolytes (compatible solutes) in its salt-out strategy for salt acclimation (Scanlan et al. 2009). Most strains of *Prochlorococcus* are thought to use glucosylglycerate and sucrose as their main osmolytes, but some LL strains are instead thought to use glycine betaine (Scanlan et al. 2009). Although these LL strains are found at deeper depths than were sampled at the high-salinity RS and MED sites, it is clear that both *Prochlorococcus* and other cyanobacteria and algae have the capacity to produce osmolytes like glycine betaine that may be important energy and nutrient sources for SAR11 in these environments.

Given that SAR11 is the most abundant organism on the planet, if osmolyte consumption is a major source of SAR11's organic carbon and nutrients, this must also be a major flux in the earth's biogeochemical cycles. It remains to be shown which osmolytes (with which elemental compositions) are produced and consumed by which organisms under diverse nutrient and salinity conditions. Our data provide some initial hints, however. For example, genes for utilization of GBT and creatine are preferentially over-represented in RS, whereas genes for utilization of DMSP and TMAO are preferentially over-represented in MED (see metabolic pathway in Sun et al. (2011)). GBT and creatine, and DMSP and TMAO, therefore, may be more commonly produced and consumed in the Red Sea and Mediterranean, respectively. Adaptive use of labile organic compounds as electron and nutrient sources will be an important area of future research in marine microbial ecology.

### Ecotype-level distribution patterns in *Prochlorococcus* and cyanophage

An open question in microbial ecology is how functional diversity covaries with phylogenetic diversity. Specifically, among a population composed of major and minor phylogenetic subtypes (e.g., two ecotypes of *Prochlorococcus*), are ecosystem-specific functional adaptations found predominantly in the major subtype of that environment, or are some adaptations found more often in the minor subtype? In other words, are the ecotype distributions of ecosystem-enriched gene clusters different from the ecotype distributions of all gene clusters in a given ecosystem and population? Our dataset, with gene cluster frequencies that can be mapped back to the ecotypes from which they originate, gives us the opportunity to address this question.

We found that most of the ecosystem-enriched gene clusters in *Prochlorococcus* and cyanophage have similar ecotype distributions to nonenriched gene clusters (Fig. 2). Relative to the average ecotype frequencies, many of the ecosystem-enriched gene clusters actually have more skewed ecotype frequencies. What these results suggest is that *Prochlorococcus* ecotypes and cyanophage types are largely cohesive. If there were rampant horizontal gene transfer bringing in adaptive genes from other ecotypes, we would expect sea-enriched gene clusters to have a different ecotype distribution than the other gene clusters. Our findings instead suggest that most of the important adaptation in gene copy number is occurring within the most dominant ecotype.

There were, however, some notable exceptions in *Prochlorococcus* where ecosystem-enriched gene clusters deviated from the ecotype frequencies of most gene clusters (Fig. 2). The high incidence of HLI-type photolyase (PRO2760) in RS, which is almost exclusively dominated by HLII genes, suggests that HLII cells in RS have acquired a HLI-type photolyase to adapt to high irradiance in the Red Sea. Interestingly, the arsenite efflux pump (PRO2832) that is implicated in the *Prochlorococcus* low-phosphorus response deviates in ecotype distribution in both MED and BATS, but in different ways: in MED it is nearly all HLII-type (the majority of gene clusters in MED are comprised mostly of HLI), but in BATS it is relatively more LL-type (there is some HLII-type, but less than most other gene clusters in BATS). This result points to a dynamic evolutionary history for arsenite efflux in low-phosphorus waters, with HLI cells acquiring an HLII gene in MED, and HLII cells acquiring a LL gene in BATS.

### Competitive strategies of oligotrophic marine microbes

The relative abundance of genetic functions among diverse environments can be used to address the ecological strategies of *Prochlorococcus*, cyanophage, and SAR11. Following Grime's CSR (competitor–stress tolerator–ruderal) strategies for plants (Grime 2001), we can ask whether a microbial group is optimized for either high growth rate under intermittently high substrate availability (competitors or ruderals) or high substrate affinity to maintain carrying capacity under consistently low substrate availability (stress tolerators). The compact genomes and small cell sizes (high surface-to-volume ratio) of *Prochlorococcus* and SAR11 are established evidence supporting a stress toleration strategy. Are the gene frequency data consistent with this strategy? In *Prochlorococcus*, we find increased selection for DNA repair in high-irradiance environments. This reflects long-term maintenance of genome fidelity, which may be more important for maintaining carrying capacity than for a boom-and-bust strategy. The phosphate ABC transport system found over-represented in *Prochlorococcus*, cyanophage, and SAR11 in the low-phosphorus BATS and MED environments is a high-affinity transporter. High substrate affinity is a hallmark of stress tolerators, which are evolved for consistently low nutrient concentrations rather than intermittent high concentrations (Prosser et al. 2007). Likewise, salinity is generally stable in open-ocean environments (Scanlan et al. 2009), and free osmolytes in higher-salinity environments like RS and MED sites are expected to be continuously present at elevated concentrations. The enhanced ability for SAR11 to oxidize osmolytes at these sites is therefore further evidence of a stress toleration strategy.

### Conclusions and future directions

The Red Sea microbial community, never before studied with metagenomics, has genetic adaptations that reflect its unique combination of physicochemical properties. Red Sea microbes resemble the North Pacific in high-light adaptation, the Mediterranean in adaptation to high salinity, and (to a lesser extent) the Mediterranean and Sargasso Seas in adaptation to low phosphorus. SAR11 populations in the Red Sea and Mediterranean point to osmolytes as important electron donors in saline waters, a potentially major biogeochemical process in the world ocean. Ecotype-scale resolution of gene frequencies among *Prochlorococcus* and cyanophage populations indicates that the predominant ecotypes in populations contribute most of the ecosystem-specific adaptation.

Going forward, the unique environment of the Red Sea will continue to inform global marine microbial processes. Investigations of microbial adaptation along the Red Sea's gradients of salinity and other physicochemical parameters will help inform, for example, the organisms producing and consuming various osmolyte species. Much of the adaptation of microbes to their local environment is conferred not at the level of gene copy number (DNA) but at transcriptional regulation (messenger RNA). Comparisons of community gene expression along day–night and seasonal axes, between populations in different seas (for example, the Red Sea and Mediterranean), will inform how evolutionary processes affect much shorter time-scales of adaptation. Finally, cultivations of *Prochlorococcus*, cyanophage, and SAR11 from the Red Sea, which are on-going in our laboratory, will allow testing of the most compelling hypotheses in controlled physiological studies.

## Supporting Information

**Data S1.** Methods.

**Table S1**. Genomes used in this study for building gene clusters.

**Table S2**. Hypervariable regions of *Prochlorococcus*, cyanophage, and SAR11 reference genomes.

**Table S3**. Complete list of gene clusters over- or under-represented in BATS, HOT, MED, or RS.

**Figure S1**. CTD traces for sampling done at RS, MED, BATS, and HOT. Representative casts are shown from KRSE2008, PROSOPE, BATS216, and HOT186 cruises, respectively. Casts were the same as those used to collect samples for DNA sequencing except MED, where the cast was made on Sept. 15, 1999 at a station near the sampling site. Temperature is shown with solid lines, and relative fluorescence (chlorophyll) is shown with dashed lines. Depths where samples were taken for pyrosequencing are marked with dotted lines

**Figure S2**. Schematic overview of the methods. (A) Assigning metagenomic reads to gene clusters. Reads from each sample were compared to GenBank-nr using BLASTX and binned as *Prochlorococcus*, cyanophage, or SAR11. Reads in each taxonomic bin were then compared to the available genomes for that taxonomic group using BLASTN and assigned to gene clusters. (B) Calculating relative normalized abundances and entropies for each gene cluster. In this example, counts for the three BATS and three HOT samples were combined. Normalized abundance was calculated by normalizing over the gene clusters for each sample. Relative normalized abundance was calculated by normalizing over the samples for each gene cluster. Shannon entropy was calculated from r.n.a. PRO1000, PRO1001, and PRO1002 are core gene clusters, while PRO2983 is a flexible gene cluster (alkaline phosphatase).

**Figure S3**. Relative abundance of 16S rRNA genes obtained from metagenomic libraries of RS, MED, BATS, and HOT. (A) Phylum-level classification for all recruited reads. (B) Genus-level classification of the phylum Proteobacteria. (C) Genus-level classification of the phylum Cyanobacteria.

**Figure S4**. Taxonomic distribution of metagenomic reads from the four datasets included in this study. Top BLAST hits to sequenced genomes are shown, with subgroup/ecotype subdivisions of the counts shown where available. Note that only SAR11 subgroups 1a and 3 are represented by genomes, so only those two subgroups are shown.

**Figure S5**. Relative normalized abundance and entropy of single-copy gene clusters (found exactly once in each genome) and non-single-copy gene clusters (found more or less than once in at least one genome) from *Prochlorococcus*, cyanophage, and SAR11 in a genomic context. Gene clusters with entropy in the bottom 15% (*Prochlorococcus*, SAR11) or 25% (cyanophage) and r.n.a. for one sea in the top or bottom 10% are marked with solid black lines. The dotted line indicates r.n.a. equal to 0.25 (i.e., equal normalized abundance across the four seas). Gray boxes indicate HVRs (Methods).

**Figure S6**. Histograms of entropy values for single-copy gene clusters (found exactly once in each genome) and non-single-copy gene clusters (found more or less than once in at least one genome) from *Prochlorococcus*, cyanophage, and SAR11. Only those gene clusters with greater than 20 hits across the four samples are shown. Note the differences in y-axis scale bars between the single-copy and non-single-copy histograms.
