# Supplementary material for: Patterns of ecological specialization among microbial populations in the Red Sea and diverse oligotrophic marine environments
Source: Ecol Evol. 2013 May 11;3(6):1780–97. doi: 10.1002/ece3.593 (PMC3686209; doi:10.1002/ece3.593)
Supplement: Supplementary file 1 [file ece30003-1780-SD1.pdf]

Supporting Information

SI Methods

Sampling, DNA extraction, and sequencing of Red Sea sample. Seawater (120 L) was collected from a depth of 50 m at the Atlantis II Deep area during the KAUST Red Sea Expedition in October 2008 (see Table 1 for details about the samples and sampling locations). Microbial content was serially fractionated on mixed-cellulose-ester filters with pore sizes of 3.0, 0.8 and 0.1 μm, respectively, using Millipore 293-mm stainless steel sanitary filter holders (Durapore, Millipore, Billerica, MA, USA). Filters were stored in sucrose lysis buffer (Rusch et al., 2007) at –20 °C for delivery to the AUC Genomics Facility in Cairo, and stored at –70 °C. DNA was isolated from the 0.1-μm filter as described by Rusch et al. (2007), with modification by the CTAB treatment outlined in the DOE Joint Genome Institute protocol (http://my.jgi.doe.gov/general/; DNA Isolation Bacterial CTAB Protocol). DNA concentration was determined using a NanoDrop3300 Fluorospectrometer (Thermo Scientific, USA) and the Quant-iT PicoGreen dsDNA Kit (Invitrogen, USA). The DNA was used to construct a GS FLX Titanium Library as recommended (Roche, Germany), and sequencing was performed on a GS FLX Pyrosequencer using the Titanium Pyrosequencing Kit (454 Life Sciences). The sequenced data set was processed using 454 GS FLX/FLX+ Data Processing Software, and de-replication was performed by removing exact replicates followed by removal of near-exact replicates (98% identity) using CD-HIT-454 (Niu et al., 2010).

Existing data sets. Pyrosequenced metagenomic data sets for surface microbial communities were obtained from previous studies of the Mediterranean deep chlorophyll maximum (Ghai et al., 2010), the Bermuda Atlantic Time Series (BATS216), and the Hawaii Ocean Time-series (HOT186) (Coleman and Chisholm, 2010). Database and source water properties are described in Table 1. Water column CTD traces are shown in Fig. S1.

Measurement of physical and chemical parameters. Physical and chemical data for the four sampling sites were acquired directly or taken from literature and online databases (Table 1). RS values are from station 106 (21.247° N, 38.298° E), cast 2 (Sept. 26, 2011) of the 2011

KAUST Red Sea Expedition; nutrient analyses were carried out at the UCSB Marine Science Institute on a 0.1-μm filtered sample from 50 m; salinity and temperature data from the CTD represent ranges from within 1 m of 50 m. MED nitrate+nitrite is from the sampling site in June 1986 (Estrada et al., 1993), and salinity and temperature values are from World Ocean Atlas (http://www.nodc.noaa.gov/) climatological means for October at 50 m. BATS and HOT values are from BATS cruise 216 (Oct. 2006) and HOT cruise 186 (Oct. 2006), with nutrient values reported from the closest available depths (ranges reported if multiple values) and salinity and temperature ranges from within 1 m of the indicated depth. BATS and HOT data retrieved from http://bats.bios.edu/ and http://hahana.soest.hawaii.edu/. Shortwave downward irradiance at the surface is from the HIRAM model at 25-km resolution (M. Dogar and G. Stenchikov, pers. comm.).

Taxonomic analysis of 16S rRNA genes in metagenomes. The abundance and distribution of 16S rRNA genes (Bacteria and Archaea) in each metagenomic library was assessed by BLASTN-based searches for 16S homologues against a GenBank-based 16S rRNA gene database. Best matches to our query sequences were counted as those that had a minimum length of 200 bp and a sequence identity of >95% to the query sequence, bit score value of >40, and an expectation value of $<10^{-5}$. Recruited reads were then taxonomically assigned based on an annotated reference database (SILVA v. 104; http://www.arb-silva.de/) using a Bayesian classifier (Wang et al., 2007) as implemented in mothur (Schloss et al., 2009) with a minimum assignment threshold of 80% (1,000 iterations). The relative abundance of each phylum (or class or order) in a sample was then expressed as the percentage of all sequence counts recruited per sample: RS_50m (631), MED_50m (1074), BATS_20m (422), BATS_50m (442), BATS_100m (382), HOT_25m (588), HOT_75m (598), and HOT_110m (502).

Assignment of metagenomic reads to taxonomic groups. The assignment of metagenomic reads to gene clusters and subsequent statistical analyses are described here and are summarized in Fig. S2. Individual, unassembled reads were first compared to the GenBank non-redundant protein database (GenBank-nr) using BLASTX (e-value $<10^{-4}$, bit score >40). Examining the top five BLASTX hits for each read, we binned reads into different taxonomic groups. If the first hit was to a sequenced SAR11, *Prochlorococcus*, cyanophage, or

Synechococcus genome, the read was placed into the respective bin. If the top hit was to any other taxonomic group, the read was assigned as “Other”. If the first hit was to an uncultured sequence, that hit was ignored and the next hit examined; if each of the top five hits for a read was uncultured, that read was assigned as “Uncultured”.

Assignment of taxon-specific reads to taxon-specific gene clusters. Reads binned as *Prochlorococcus*, cyanophage, or SAR11 were assigned to gene clusters. Genomes (proteomes) were downloaded from GenBank (Table S1). Gene clusters were built using all vs. all BLASTP output with the program OrthoMCL (Li et al., 2003). Singletons (proteins not clustering with any other protein) were designated as their own gene clusters. Gene clusters were then categorized as single-copy (i.e., each genome has one and only one copy of the gene in its genome) or non-single-copy. This is similar to the distinction between core and non-core (flexible) gene clusters, with the difference that core genes found in more than one copy are classified as non-single-copy. For assigning reads to gene clusters (Fig. S2a), reads were compared to *Prochlorococcus*, cyanophage, or SAR11 genomes using BLASTN (bit score >40, alignment length >40 for *Prochlorococcus* and cyanophage, alignment length >30 for SAR11 (Coleman and Chisholm, 2010)). If a read hit two genes in the same genome, the gene with the longer alignment was used. Reads were then assigned to gene clusters as follows: For each read, the top three hits among the genomes had to be to genes in the same gene cluster, if the gene cluster had three or more entries. If the gene cluster had only one or two entries, only the top one or two hits had to be to that gene cluster. If neither condition was met, the read was not counted as belonging to any gene cluster and therefore was not included in the analysis.

Calculation of relative normalized gene cluster abundances across seas. The gene cluster abundance data were analyzed separately for five different subsets of the eight metagenomic samples: (1) all eight samples with BATS and HOT data summed, (2) all eight samples with BATS and HOT data separated, (3) mixed layer samples (BATS 20 m, HOT 25 m), (4) below mixed layer samples (BATS 50 m, HOT 75 m, RS 50 m), and (5) deep chlorophyll maximum samples (BATS 100 m, HOT 110 m, MED 50 m). Unless otherwise noted, the results presented were derived from the first method (Fig. S2b). First, gene clusters with total read counts of 20 or less across all samples were removed. Read counts for each gene cluster and

sample were then normalized for each sample to the total number of recruited reads in all gene clusters. These normalized counts were then further normalized for each gene cluster across the samples. We call the resulting metric "relative normalized abundance" (r.n.a.). Shannon entropy was used to differentiate gene clusters with skewed abundance distributions across the samples from gene clusters with uniform abundance distributions. If (p_1, p_2, p_3, p_4) are the r.n.a.s for a gene cluster in samples 1–4 (e.g. RS, MED, BATS, and HOT), the Shannon entropy is computed as $-\sum_{i=1}^{4} p_i log(p_i)$, where $p_i log(p_i)$ is set equal to 0 when $p_i = 0$. For example, a gene cluster with a purely uniform distribution across the four samples would have an r.n.a. of 0.25 in each of the four samples and a maximal entropy value (1.3863); a gene cluster with the most skewed distribution possible would have an r.n.a. of 1 in one sample and 0 in the three other samples, with an entropy value of 0. Using these calculated r.n.a.s and entropies, gene clusters were identified that were over- or under-represented in one of the samples. To be considered over- or under-represented, gene clusters were required to have an r.n.a. for that sample in the top or bottom 10% of gene clusters, an entropy in the lowest 15% (*Prochlorococcus* and SAR11) or 25% (cyanophage), and a total read count across all samples in the top 75%.

Ecotype distributions of reads assigned to gene clusters. Relative contributions of different *Prochlorococcus* or cyanophage ecotypes to read counts for each gene cluster in each sea were calculated using the top BLASTN hits from the above analysis. For each read assigned to a gene cluster, the top BLASTN hit and the ecotype or phage type classifications in Table S1 were used to assess the relative contributions of those ecotypes or phage types to the total pool of reads belonging to that gene cluster. Only gene clusters with greater than 20 total reads across the four seas were included in the analysis. Gene clusters were considered outliers if any one of the ecotypes had a relative frequency greater than $q_3 + 1.5(q_3 - q_1)$ or less than $q_1 - 1.5(q_3 - q_1)$, where q_1 and q_3 are the 25th and 75th percentiles, respectively. As an additional measure of outlierness, ecotype distributions were compared using Kullback–Leibler distances (Kullback and Leibler, 1951), and those gene clusters with larger KL distances from the mean than 80% of the non-over-represented gene clusters were considered outliers.

Genomic context plots and hypervariable regions. Gene cluster r.n.a. values were plotted versus gene position in reference genomes. Across four samples, an r.n.a. of 0.25 in each sample represents a perfectly evenly distributed gene cluster; a deviation of values from 0.25 represents over- or under-representation among the four seas. Hypervariable regions (HVRs) in the reference genomes were identified using MUMmer (Delcher et al., 2002) with the following commands: nucmer -minmatch 10 -breaklen 1200 -maxgap 1000 -mincluster 220; show-coords -L 2400. Gaps between alignments were called HVRs if they measured at least 8,000 bp. Gaps in *Prochlorococcus* MIT9301, SAR11 HTCC7211, and cyanophage S-SM2 (our reference genomes) were mapped to *Prochlorococcus* MIT9312, SAR11 HTCC1062, and cyanophage S-RSM4 (genomes with published HVRs) using BLASTN and Artemis Comparison Tool (Carver et al., 2005).

Clustering of seas by gene cluster abundance patterns. Hierarchical clustering was done using the program AGNES (Kaufman and Rousseeuw, 2005) with Kullback–Leibler distances (Kullback and Leibler, 1951), implemented in R with the cluster library (http://www.R-project.org/). For reference, the average Kullback–Leibler distance is defined as $\sum(p_i - q_i)log(p_i/q_i)$ for relative abundances p and q. To cluster the four seas, hierarchical clustering was performed on the normalized abundances for each sample, using only those gene clusters with entropy in the lowest 25% and a total read count across the four seas in the top 75%.

SI Tables

Table S1: Genomes used in this study for building gene clusters. Group/genus and ecotype/subgroup designations were used to classify the metagenomic reads and interpret gene cluster abundances among metagenomic data sets. Hosts on which phage strains were isolated are indicated in parentheses: *Pro.*, *Prochlorococcus*; *Syn.*, *Synechococcus*.

Group/Genus	Strain	Ecotype/Subgroup	Accession no.
Prochlorococcus	MED4	High-light I	NC_005072
	MIT9515	High-light I	NC_008817
	AS9601	High-light II	NC_008816
	MIT9202	High-light II	NZ_DS999537 (NZ_ACDW00000000.scaffold)
	MIT9215	High-light II	NC_009840
	MIT9301	High-light II	NC_009091
	MIT9312	High-light II	NC_007577
	NATL1A	Low-light I	NC_008819
	NATL2A	Low-light I	NC_007335
	SS120	Low-light II	NC_005042
	MIT9211	Low-light III	NC_009976
	MIT9303	Low-light IV	NC_008820
	MIT9313	Low-light IV	NC_005071
Cyanophage	P-SS2	Siphovirus (*Pro.*)	NC_013021
	P-HM1	T4-like (*Pro.*)	NC_015280
	P-HM2	T4-like (*Pro.*)	NC_015284
	P-RSM4	T4-like (*Pro.*)	NC_015283
	P-SSM2	T4-like (*Pro.*)	NC_006883
	P-SSM4	T4-like (*Pro.*)	NC_006884
	P-SSM7	T4-like (*Pro.*)	NC_015290
	S-PM2	T4-like (*Syn.*)	NC_006820
	S-RSM4	T4-like (*Syn.*)	NC_013085
	S-ShM2	T4-like (*Syn.*)	NC_015281
	S-SM1	T4-like (*Syn.*)	NC_015282
	S-SM2	T4-like (*Syn.*)	NC_015279
	S-SSM5	T4-like (*Syn.*)	NC_015289
	S-SSM7	T4-like (*Syn.*)	NC_015287
	Syn1	T4-like (*Syn.*)	NC_015288
	Syn19	T4-like (*Syn.*)	NC_015286
	Syn33	T4-like (*Syn.*)	NC_015285
	Syn9	T4-like (*Syn.*)	NC_008296
	P-SSP7	T7-like (*Pro.*)	NC_006882
	P60	T7-like (*Syn.*)	NC_003390
	Syn5	T7-like (*Syn.*)	NC_009531
SAR11	HTCC1002	Subgroup 1a	NZ_CH724130 (NZ_AAPV00000000.scaffold)
	HTCC1062	Subgroup 1a	NC_007205
	HTCC7211	Subgroup 1a	NZ_DS995298 (NZ_ABVS00000000.scaffold)
	HIMB114	Subgroup 3	NZ_GG704918 (NZ_ADAC00000000.scaffold)
	IMCC9063	Subgroup 3	NC_015380

Table S2: Hypervariable regions of *Prochlorococcus*, cyanophage, and SAR11 reference genomes. HVRs were identified using whole-genome alignments as described in SI Methods. Analogous HVRs in published genomes are listed where available.

	Start (bp)	End (bp)	Size (bp)	Published
Prochlorococcus MIT9301	343734	355255	11522	MIT9312 ISL1 (Coleman et al., 2006)
	605338	632741	27404	MIT9312 ISL2 (Coleman et al., 2006)
	1053342	1105863	52522	MIT9312 ISL3 (Coleman et al., 2006)
	1163685	1218926	55242	MIT9312 ISL4 (Coleman et al., 2006)
	1321145	1352730	31586	MIT9312 ISL5 (Coleman et al., 2006)
Cyanophage S-SM2	140106	172954	32849	S-RSM4 HVR (Millard et al., 2009)
SAR11 HTCC7211	1	44622	44622	HTCC1062 HVR2 (Wilhelm et al., 2007)
	532501	543356	10856	
	671398	689605	18208	
	708046	723993	15948	HTCC1062 HVR4 (Wilhelm et al., 2007)
	740276	766078	25803	HTCC1062 HVR3 (Wilhelm et al., 2007)
	800069	830923	30855	
	965996	991473	25478	
	994211	1004253	10043	
	1073258	1083636	10379	
	1141286	1149585	8300	
	1164921	1177927	13007	
	1198003	1208029	10027	
	1246586	1263657	17072	
	1270100	1302131	32032	
	1332746	1351965	19220	
	1420357	1456888	36532	HTCC1062 HVR2 (Wilhelm et al., 2007)

Table S3: Complete list of gene clusters over- or under-represented in BATS, HOT, MED, or RS. For each gene cluster, relative normalized abundance for each sample, entropy, number of reads mapping, proposed function, total number of copies among the genomes, and a representative locus tag is given. Analyses of five separate subsets of the eight metagenomic samples are presented: (1) all eight samples with BATS and HOT data summed, (2) all eight samples with BATS and HOT data separated, (3) mixed layer samples (BATS 20 m, HOT 25 m), (4) sub-mixed layer samples (BATS 50 m, HOT 75 m, RS 50 m), and (5) deep chlorophyll maximum samples (BATS 100 m, HOT 110 m, MED 50 m). Attached file: Thompson_TableS3.xlsx

SI Figures

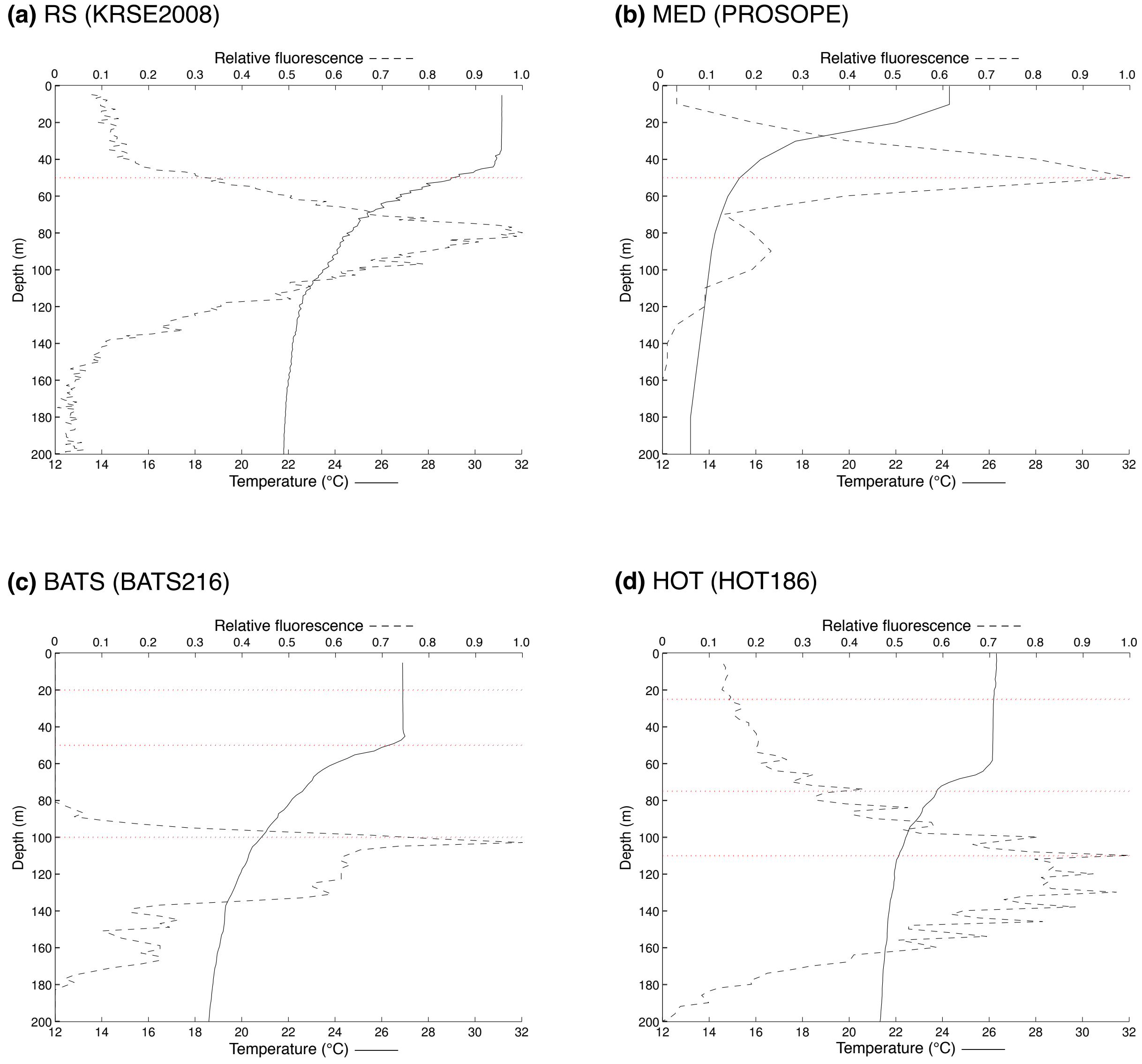

Figure S1: CTD traces for sampling done at RS, MED, BATS, and HOT. Representative casts are shown from KRSE2008, PROSOPE, BATS216, and HOT186 cruises, respectively. Casts were the same as those used to collect samples for DNA sequencing except MED, where the cast was made on Sept. 15, 1999 at a station near the sampling site. Temperature is shown with solid lines, and relative fluorescence (chlorophyll) is shown with dashed lines. Depths where samples were taken for pyrosequencing are marked with dotted lines.

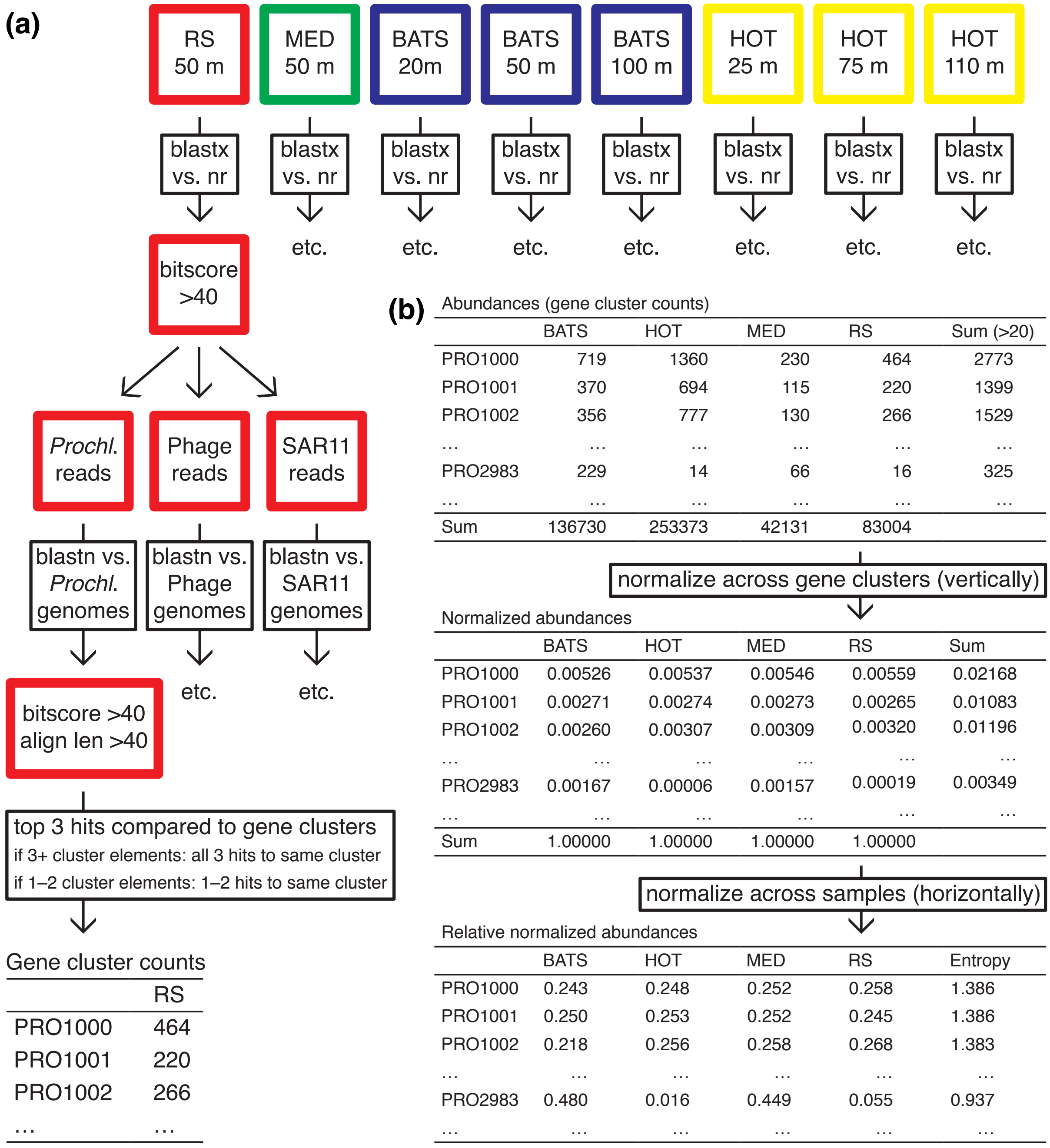

Gene cluster counts

	RS
PRO1000	464
PRO1001	220
PRO1002	266
…	…

Abundances (gene cluster counts)

	BATS	HOT	MED	RS	Sum (>20)
PRO1000	719	1360	230	464	2773
PRO1001	370	694	115	220	1399
PRO1002	356	777	130	266	1529
…	…	…	…	…	…
PRO2983	229	14	66	16	325
…	…	…	…	…	…
Sum	136730	253373	42131	83004	

Normalized abundances

	BATS	HOT	MED	RS	Sum
PRO1000	0.00526	0.00537	0.00546	0.00559	0.02168
PRO1001	0.00271	0.00274	0.00273	0.00265	0.01083
PRO1002	0.00260	0.00307	0.00309	0.00320	0.01196
…	…	…	…	…	…
PRO2983	0.00167	0.00006	0.00157	0.00019	0.00349
…	…	…	…	…	…
Sum	1.00000	1.00000	1.00000	1.00000	

Relative normalized abundances

	BATS	HOT	MED	RS	Entropy
PRO1000	0.243	0.248	0.252	0.258	1.386
PRO1001	0.250	0.253	0.252	0.245	1.386
PRO1002	0.218	0.256	0.258	0.268	1.383
…	…	…	…	…	…
PRO2983	0.480	0.016	0.449	0.055	0.937
…	…	…	…	…	…

Figure S2: Schematic overview of the methods. (a) Assigning metagenomic reads to gene clusters. Reads from each sample were compared to GenBank-nr using BLASTX and binned as *Prochlorococcus*, cyanophage, or SAR11. Reads in each taxonomic bin were then compared to the available genomes for that taxonomic group using BLASTN and assigned to gene clusters. (b) Calculating relative normalized abundances and entropies for each gene cluster. In this example, counts for the three BATS and three HOT samples were combined. Normalized abundance was calculated by normalizing over the gene clusters for each sample. Relative normalized abundance was calculated by normalizing over the samples for each gene cluster. Shannon entropy was calculated from r.n.a. PRO1000, PRO1001, and PRO1002 are core gene clusters, while PRO2983 is a flexible gene cluster (alkaline phosphatase).

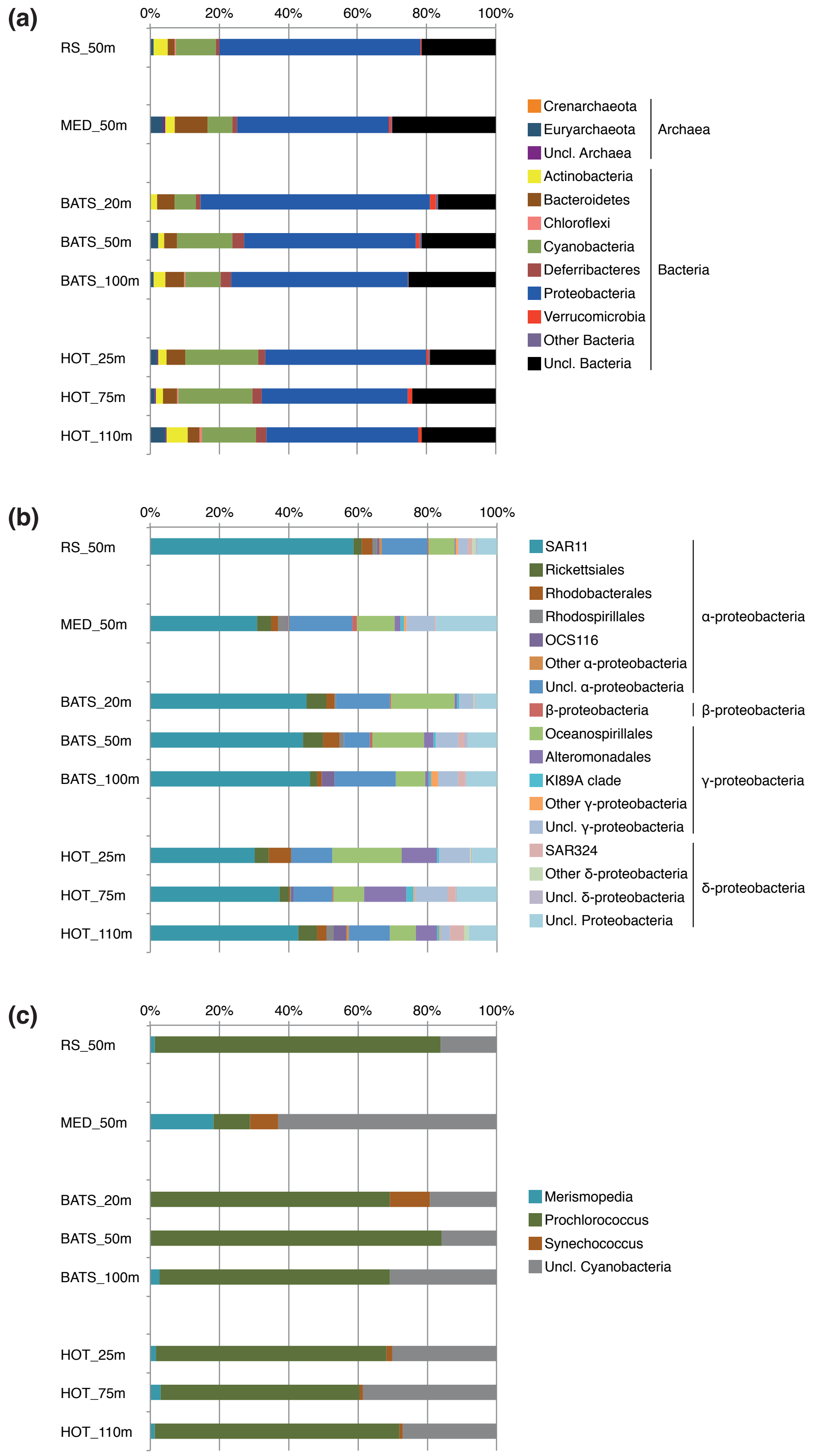

Figure S3: Relative abundance of 16S rRNA genes obtained from metagenomic libraries of RS, MED, BATS, and HOT. (a) Phylum-level classification for all recruited reads. (b) Genus-level classification of the phylum Proteobacteria. (c) Genus-level classification of the phylum Cyanobacteria.

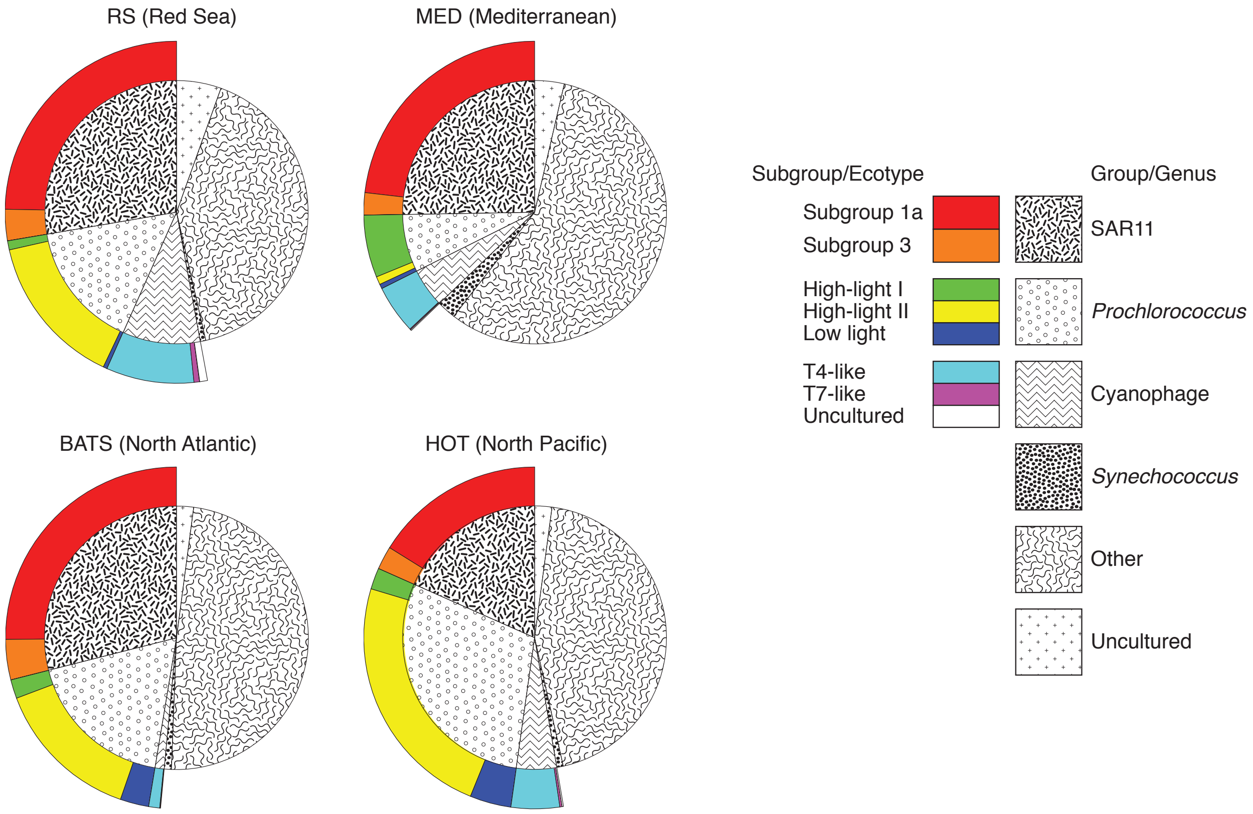

Figure S4: Taxonomic distribution of metagenomic reads from the four data sets included in this study. Top BLAST hits to sequenced genomes are shown, with subgroup/ecotype subdivisions of the counts shown where available. Note that only SAR11 subgroups 1a and 3 are represented by genomes, so only those two subgroups are shown.

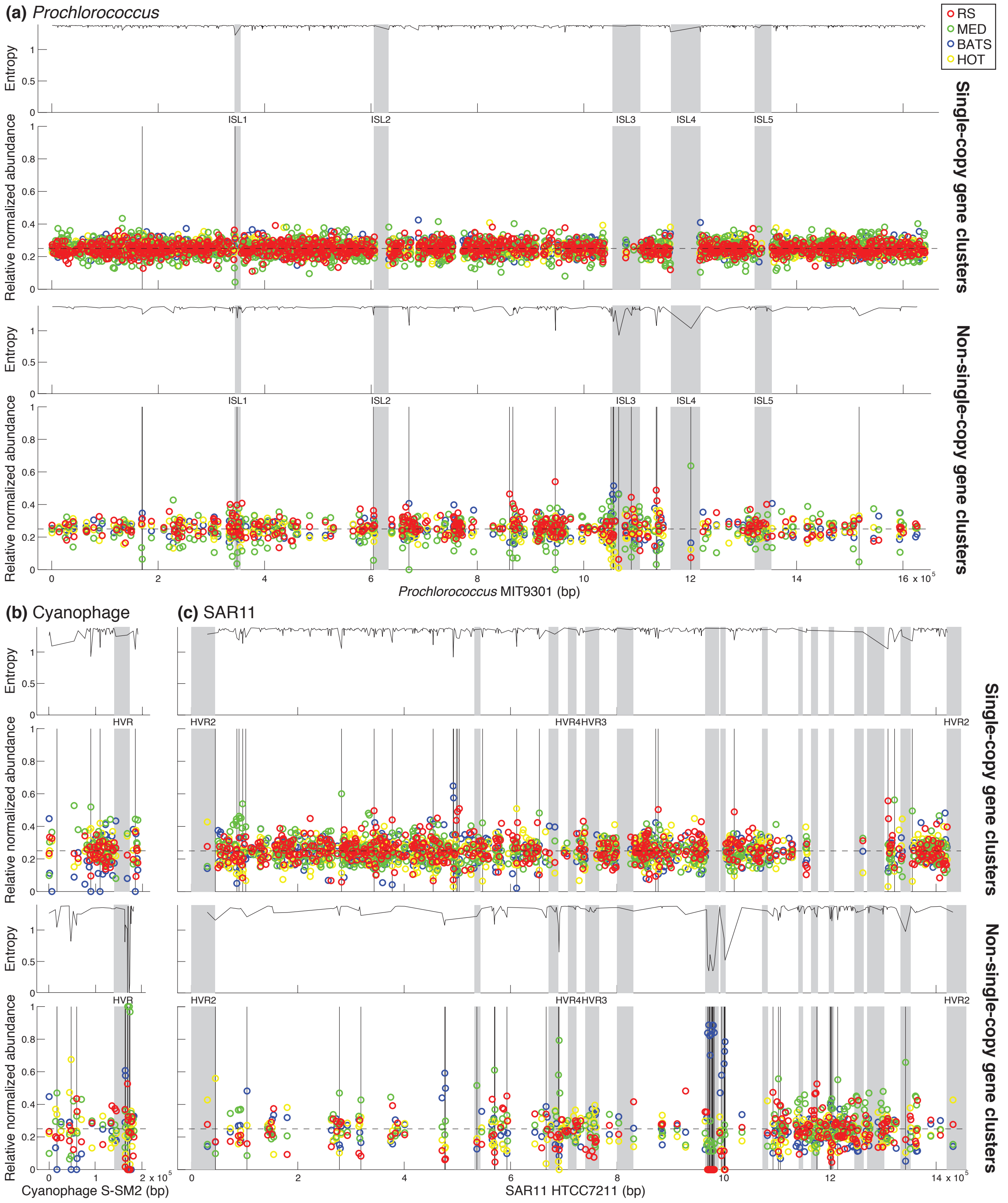

Figure S5: Relative normalized abundance and entropy of single-copy gene clusters (found exactly once in each genome) and non-single-copy gene clusters (found more or less than once in at least one genome) from *Prochlorococcus*, cyanophage, and SAR11 in a genomic context. Gene clusters with entropy in the bottom 15% (*Prochlorococcus*, SAR11) or 25% (cyanophage) and r.n.a. for one sea in the top or bottom 10% are marked with solid black lines. The dotted line indicates r.n.a. equal to 0.25 (i.e., equal normalized abundance across the four seas). Gray boxes indicate HVRs (Methods).

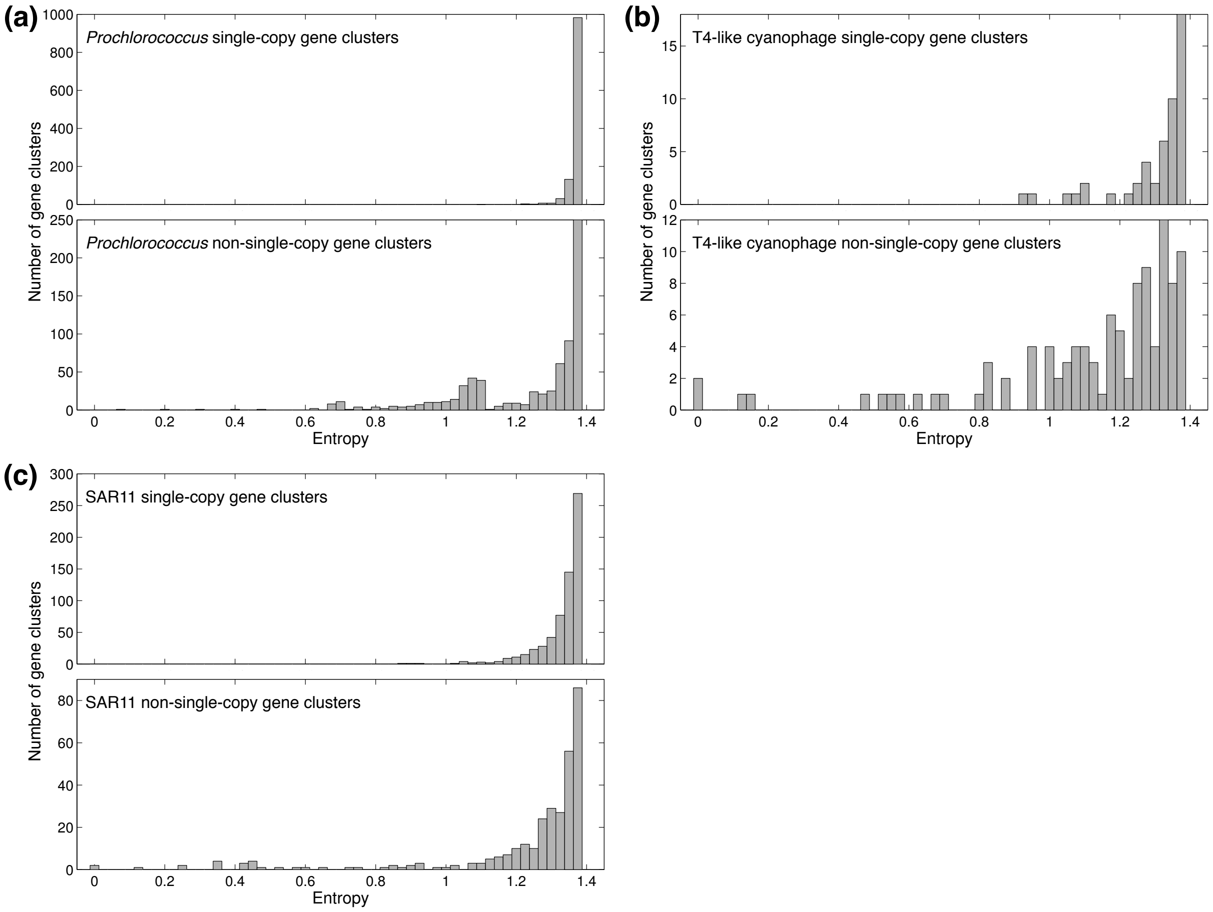

Figure S6: Histograms of entropy values for single-copy gene clusters (found exactly once in each genome) and non-single-copy gene clusters (found more or less than once in at least one genome) from *Prochlorococcus*, cyanophage, and SAR11. Only those gene clusters with greater than 20 hits across the four samples are shown. Note the differences in y-axis scale bars between the single-copy and non-single-copy histograms.

References

Carver, T. J., Rutherford, K. M., Berriman, M., Rajandream, M.-A., Barrell, B. G., & Parkhill, J. (2005). ACT: the Artemis Comparison Tool. *Bioinformatics*, 21(16):3422–3423.

Coleman, M. L. & Chisholm, S. W. (2010). Ecosystem-specific selection pressures revealed through comparative population genomics. *Proc Natl Acad Sci USA*, 107(43):18634–18639.

Coleman, M. L., Sullivan, M. B., Martiny, A. C., Steglich, C., Barry, K., DeLong, E. F., & Chisholm, S. W. (2006). Genomic islands and the ecology and evolution of Prochlorococcus. *Science*, 311(5):1768–1770.

Delcher, A. L., Phillippy, A., Carlton, J., & Salzberg, S. L. (2002). Fast algorithms for large-scale genome alignment and comparison. *Nucleic Acids Res*, 30(11):2478–2483.

Estrada, M., Marrasé, C., Latasa, M., Berdalet, E., Delgado, M., & Riera, T. (1993). Variability of deep chlorophyll maximum characteristics in the northwestern Mediterranean. *Mar Ecol Prog Ser*, 92(3):289–300.

Ghai, R., Martin-Cuadrado, A.-B., Molto, A. G., Heredia, I. G., Cabrera, R., Martin, J., Verdú, M., Deschamps, P., Moreira, D., López-García, P., Mira, A., & Rodríguez-Valera, F. (2010). Metagenome of the Mediterranean deep chlorophyll maximum studied by direct and fosmid library 454 pyrosequencing. *ISME J*, 4(9):1154–1166.

Kaufman, L. & Rousseeuw, P. J. (2005). *Finding Groups in Data: An Introduction to Cluster Analysis*. Wiley Series in Probability and Statistics. Wiley-Interscience.

Kullback, S. & Leibler, R. A. (1951). On information and sufficiency. *Ann Math Statist*, 22(1):79–86.

Li, L., Stoeckert, C. J., & Roos, D. S. (2003). OrthoMCL: identification of ortholog groups for eukaryotic genomes. *Genome Res*, 13(9):2178–2189.

Millard, A. D., Zwirglmaier, K., Downey, M. J., Mann, N. H., & Scanlan, D. J. (2009). Comparative genomics of marine cyanomyoviruses reveals the widespread occurrence of

Synechococcus host genes localized to a hyperplastic region: implications for mechanisms of cyanophage evolution. *Environ Microbiol*, 11(9):2370–2387.

Niu, B., Fu, L., Sun, S., & Li, W. (2010). Artificial and natural duplicates in pyrosequencing reads of metagenomic data. *BMC Bioinformatics*, 11:187–187.

Rusch, D. B., Halpern, A. L., Sutton, G., Heidelberg, K. B., Williamson, S., Yooseph, S., Wu, D., Eisen, J. A., Hoffman, J. M., Remington, K., Beeson, K., Tran, B., Smith, H., Baden-Tillson, H., Stewart, C., Thorpe, J., Freeman, J., Andrews-Pfannkoch, C., Venter, J. E., Li, K., Kravitz, S., Heidelberg, J. F., Utterback, T., Rogers, Y.-H., Falcón, L. I., Souza, V., Bonilla-Rosso, G., Eguiarte, L. E., Karl, D. M., Sathyendranath, S., Platt, T., Bermingham, E., Gallardo, V., Tamayo-Castillo, G., Ferrari, M. R., Strausberg, R. L., Nealson, K., Friedman, R., Frazier, M., & Venter, J. C. (2007). The Sorcerer II Global Ocean Sampling expedition: northwest Atlantic through eastern tropical Pacific. *PLoS Biol*, 5(3):e77.

Schloss, P. D., Westcott, S. L., Ryabin, T., Hall, J. R., Hartmann, M., Hollister, E. B., Lesniewski, R. A., Oakley, B. B., Parks, D. H., Robinson, C. J., Sahl, J. W., Stres, B., Thallinger, G. G., Van Horn, D. J., & Weber, C. F. (2009). Introducing mothur: open-source, platform-independent, community-supported software for describing and comparing microbial communities. *Appl Environ Microbiol*, 75(23):7537–7541.

Wang, Q. Q., Garrity, G. M. G., Tiedje, J. M. J., & Cole, J. R. J. (2007). Naive Bayesian classifier for rapid assignment of rRNA sequences into the new bacterial taxonomy. *Appl Environ Microbiol*, 73(16):5261–5267.

Wilhelm, L. J., Tripp, H. J., Givan, S. A., Smith, D. P., & Giovannoni, S. J. (2007). Natural variation in SAR11 marine bacterioplankton genomes inferred from metagenomic data. *Biol Direct*, 2:27.
